# Endozoicomonadaceae symbiont in gills of *Acesta* clam encodes genes for essential nutrients and polysaccharide degradation

**DOI:** 10.1093/femsec/fiab070

**Published:** 2021-05-14

**Authors:** Sigmund Jensen, Jeremy A Frank, Magnus Ø Arntzen, Sébastien Duperron, Gustav Vaaje-Kolstad, Martin Hovland

**Affiliations:** Department of Chemistry, Biotechnology and Food Science, Norwegian University of Life Sciences, PO Box 5003, 1432 Ås, Norway; Department of Chemistry, Biotechnology and Food Science, Norwegian University of Life Sciences, PO Box 5003, 1432 Ås, Norway; Department of Chemistry, Biotechnology and Food Science, Norwegian University of Life Sciences, PO Box 5003, 1432 Ås, Norway; UMR 7245 Muséum National d'Histoire Naturelle/CNRS Molécules de Communication et Adaptation des Micro-organismes and Institut Universitaire de France, CP39, 12 rue Buffon, F-75231 Paris Cedex 05, France; Department of Chemistry, Biotechnology and Food Science, Norwegian University of Life Sciences, PO Box 5003, 1432 Ås, Norway; Department of Biology, University of Bergen, PO Box 7803, 5020 Bergen, Norway; Centre for Geobiology, University of Bergen, PO Box 7803, 5020 Bergen, Norway

**Keywords:** Reef; *Acesta excavata*, Endozoicomonadaceae, genome, polysaccharide, degradation

## Abstract

Gammaproteobacteria from the family Endozoicomonadaceae have emerged as widespread associates of dense marine animal communities. Their abundance in coral reefs involves symbiotic relationships and possibly host nutrition. We explored functions encoded in the genome of an uncultured Endozoicomonadaceae ‘*Candidatus* Acestibacter aggregatus’ that lives inside gill cells of large *Acesta excavata* clams in deep-water coral reefs off mid-Norway. The dominance and deep branching lineage of this symbiont was confirmed using 16S rRNA gene sequencing and phylogenomic analysis from shotgun sequencing data. The 4.5 Mb genome binned in this study has a low GC content of 35% and is enriched in transposon and chaperone gene annotations indicating ongoing adaptation. Genes encoding functions potentially involved with the symbiosis include ankyrins, repeat in toxins, secretion and nutritional systems. Complete pathways were identified for the synthesis of eleven amino acids and six B-vitamins. A minimal chitinolytic machinery was indicated from a glycosyl hydrolase GH18 and a lytic polysaccharide monooxygenase LPMO10. Expression of the latter was confirmed using proteomics. Signal peptides for secretion were identified for six polysaccharide degrading enzymes, ten proteases and three lipases. Our results suggest a nutritional symbiosis fuelled by enzymatic products from extracellular degradation processes.

## Teaser

Clam hosted bacteria inform on deep-water coral reef functioning.

## INTRODUCTION

Unexpectedly dense and thriving animal communities occur on the deep seafloor at hydrothermal vents and at cold seeps (Dubilier, Bergin and Lott 2008), in sunken wood (Distel, DeLong and Waterbury 1991) and in whale bones (Goffredi *et al*. 2014). These communities vividly exemplify how nutritional relationships with bacteria can utilize a rich but rather inaccessible carbon and energy resource. Feeding is supported from within the bacterial genomes by genes encoding proteins for the utilization of sulfide, methane, hydrogen, carbon dioxide and recalcitrant organic materials like the structural polysaccharide cellulose. Some of the best known marine heterotrophic symbionts are intracellular Gammaproteobacteria of the orders Cellvibrionales in Teredinidae bivalves (shipworms) that degrade wood (Distel, DeLong and Waterbury 1991; Yang *et al*. 2009; O'Connor *et al*. 2014; Sabbadin *et al*. 2018) and Oceanospirillales in Siboglinidae polychaetes (boneworms) that probably degrade collagen (Goffredi *et al*. 2014). Exploring symbiotic bacterial genomes not only provide insight into community function. In addition may enzyme technology be provided for the improved degradation of organic matter like polysaccharides to more useful monomeric sugars (Horn *et al*. 2012; Hemsworth *et al*. 2015). Oceanospirillales bacteria can excrete hydrolytic enzymes and degrade complex organic compounds (Garrity, Bell and Lilburn 2005) but are not well understood. As symbionts they may also compete for nutrients and degrade host tissue with the very same enzymes if environmental conditions change and food becomes scarce.

In mollusc hosts, Oceanospirillales can inhabit gill tissue, as first observed with chemoautotrophic *Bathymodiolus* mussels at hydrothermal vents on the Pacific-Antarctic Ridge, the Mid-Atlantic Ridge and in cold seeps in the Gulf of Mexico (Zielinski *et al*. 2009). Similar associations exist with gills of *Alviniconcha* snails at hydrothermal vents in the eastern Lau spreading centre (Beinart *et al*. 2014) and with *Phacoides pectinatus* clams in sulfur-rich seagrass bed sediments of Wildcat Cove mangroves in Florida (Lim *et al*. 2019). A metagenome-assembled genome (MAG) and transcript sequences from the *Phacoides* symbiont encoded transposons, toxin secretion, synthesis of amino acids, B-vitamins and fatty acid degradation (Lim *et al*. 2019). The MAG's 16S rRNA gene sequence affiliated with the genus *Kistimonas* and a bin40 MAG from the golden tube sponge *Aplysina aerophoba* in coastal Slovenia (Slaby *et al*. 2017). MAGs from the related genus *Endozoicomonas* associated with tropical corals like *Stylophora, Pocillopora* and *Acropora* in the Red Sea (Neave *et al*. 2017) and *Porites* in the Great Barrier Reef (Robbins *et al*. 2019). *Endozoicomonas* visualized in tissue of the *Stylophora* were observed forming scattered cyst-like aggregations near the coral's Symbiodiniaceae (Bayer *et al*. 2013). The Symbiodiniaceae are intracellular dinoflagellates (algae) that supply many tropical invertebrates with photosynthates such as glucose (Burriesci, Raab and Pringle 2012) which *Endozoicomonas* may use for essential nutrient synthesis (Neave *et al*. 2017). The first characterized and cultured *Endozoicomonas* was isolated from the gastrointestinal tract of an algae sap sucking *Elysia* slug, in coastal Japan (Kurahashi and Yokota 2007). All these documented symbionts and more marine invertebrate-associated Oceanospirillales belong to a single phylogenetic clade, recently proposed as the family Endozoicomonadaceae (Bartz *et al*. 2018). Although their taxonomy was reclassified (Liao *et al*. 2020), it has been debated (Neave *et al*. 2016). Nevertheless, to provide a convenient delineation of the host associates this study uses the taxon Endozoicomonadaceae. Their genomes are relatively large, 4.0–6.3 MB (Neave et al. 2014, 2017; Ding *et al*. 2016; Bartz *et al*. 2018), encoding phenotypes that potentially range from parasitic consumers of host tissue and cell nuclei to beneficial symbionts that assist in metabolism (Beinart *et al*. 2014).

A novel Endozoicomonadaceae association was observed involving the bacterium ‘*Candidatus* Acestibacter aggregatus’ and its host, the clam *Acesta excavata* (family Limidae). Distinct from other Endozoicomonadaceae based on 16S rRNA gene sequence similarity (>7% divergence) and forming intracellular cyst-like aggregations localized with no recognized autotroph, ‘*Ca*. A. aggregatus’ was found dominating the gill microbiome of all previously investigated *A. excavata* clams from deep-water coral reefs on the continental margin and in a fjord rock wall, mid-Norway (Jensen *et al*. 2010). Its closest known relatives are the *Phacoides* and *Aplysina* hosted *Kistimonas* (Slaby *et al*. 2017; Lim *et al*. 2019) and the *Stylophora* hosted *Endozoicomonas* (Bayer *et al*. 2013; Neave *et al*. 2017; Robbins *et al*. 2019). The clam *A. excavata* is widespread along the East Atlantic continental margin and has been found at depths of 33–3200 m (Vokes *et al*. 1963). The clam is characterized by an orange- to red-colored body of large size (up to 20 cm), large gill area (up to 240 cm^2^) and very high filtration rate (>100 L/h) for feeding on dissolved and particulate organic matter such as unicellular algae (Järnegren and Altin 2006). Its food is filtered from the surrounding water masses similar to other suspension feeding bivalves. Their guts appear of similar size in deep- and shallow-water relatives (Allen 1983). In the reef environments off mid-Norway, *A. excavata* is provided with a diet of plankton (Thiem *et al*. 2006; Jensen *et al*. 2015) and seasonally photosynthesized carbon from about 2 tons marine snow/km^2^/year plus particles resuspended from the sediment (Schlüter *et al*. 2000).

Less easily digested matter is likely to accumulate by water depth due to the preferential use of labile compounds and metabolic degradation processes generating recalcitrant compounds (Gottschalk 1988; Bergauer *et al*. 2018). Near the seafloor, the marine snow mixes with the dissolved and particulate matter from lateral transport and pore water, elevating bottom water nutrient concentrations (Hovland, Jensen and Indreiten 2012; Burdige and Komada 2015; Maier *et al*. 2020). We hypothesize that ‘*Ca*. A. aggregatus’ participates in the enzymatic degradation of the more recalcitrant plankton-derived polymers to provide cleavage products for host nutrition from material such as cell walls and exoskeletons, remnants and detritus. There are few genomes available to document the enzymatic capabilities of marine heterotrophic symbionts and to our knowledge none representing Endozoicomonadaceae from deep-water coral reef ecosystems. In the context of the poorly understood functions that underpin these reefs, we investigated the uncultured ‘Ca. A. aggregatus’ using amplicon and shotgun genome sequencing supported by proteomics.

## MATERIALS AND METHODS

### Sampling and handling

*A. excavata* clams were collected by remotely operated vehicles (ROVs) diving to deep-water coral reefs in hydrocarbon fields at fishing ground Haltenbanken, mid-Norwegian continental margin. The ROVs collected ∼15 clams from 317 m depth (65° 01′ N, 06° 32′ E) in the Kristin field October 2004 and from 369 m depth (65° 40′ N, 07° 32′ E) in the Skarv field June 2012 (Hovland 2008). The clams were put into two sampling baskets carried by the ROV. In total 16 clams were analysed, 13 by gill DNA sequencing and three more by gill and gut proteomics. All collected clams appeared as healthy adults that resembled a previous collection (Jensen *et al*. 2010). Onboard the support vessel, the bivalves were immediately frozen (−18°C) and transported to the University of Bergen where they were stored at −80°C prior to transport (on dry ice) and storage at −18°C at the Norwegian University of Life Sciences.

### Screening for ‘*Ca*. A. aggregatus’

Average sized clams (about 13 × 9 cm, 150 g) were thawed in a cold room (∼4°C) for a few hours to overnight and dissected for gill tissues that were first screened for the presence of ‘*Ca*. A. aggregatus’. Following a rinse in 5 mL PBS (pH 7.2), the gill filament samples were crushed under a cover slip or extracted for DNA. Under the microscope (Leitz Laborlux), fields were investigated for structures resembling aggregates and individual bacteria (Figure S1, Supporting Information). The number of aggregates per clam was estimated per field (ca 50 µm × 50 µm) and multiplied by gill area (Järnegren and Altin 2006). Gill samples extracted for DNA (explained below) were PCR amplified with primers 27f/1492r (Lane *et al*. 1991) targeting bacterial 16S rRNA genes. The amplicons were cut with *Sal*I, restriction fragments sorted by length and the profiles compared with a previously cloned 16S rRNA gene sequence from ‘*Ca*. A. aggregatus’ (Figure S1, Supporting Information; Supplementary Text).

### DNA extraction

DNA was extracted from gill tissues using the Qiamp DNA mini kit (Qiagen, Germany). For genome sequencing, ten extractions were pooled from a single Skarv specimen (prefixed Ae24) and RNA was removed using 2 mg/mL Ribonuclease A (Sigma-Aldrich, St. Louis, MO). Following precipitation at −20°C overnight in 0.3 M sodium acetate (pH 5.2) and ethanol (96%), the DNA was harvested by centrifugation and washed in 70% ice cold ethanol (Sambrook and Russel 2001). A sample (1 µg) was enriched using the NEBNext microbiome DNA enrichment kit (New England BioLabs, Ipswich, MA, USA). The kit is intended to select non-methylated bacterial DNA over methylated eukaryal DNA and was chosen instead of cell fractionation (Supplementary Text). Enriched DNA was purified with Agencourt Ampure XP beads (Beckman-Coulter, Beverly, MA, USA) and assessed using agarose gel electrophoresis (Sambrook and Russel 2001), a Qubit fluorometer (Invitrogen, Life Technologies, Carlsbad, CA) and a Nanodrop spectrophotometer (Thermo Fisher Scientific, Waltham, MA, USA; Figure S1, Supporting Information).

### 16S rRNA gene and genome sequencing

The 16S rRNA gene content from 13 clams was profiled using the Nextera XT Index kit for MiSeq sequencing (Illumina Inc, San Diego, CA, USA). Following the protocol for 16S rRNA gene sequencing, PCR reactions (25 µL) combined 6–15 ng template DNA, 0.2 µM bacterial and archaeal primers 341f/805r (Takahashi *et al*. 2014) and the KAPA HiFi HotStart mix containing iProof High-Fidelity polymerase (Bio-Rad, Hercules CA). Reactions were performed on a Mastercycler Gradient machine (Eppendorf, Hamburg, Germany), with 95°C for 3 min (denaturation) followed by 25 cycles of 95°C for 30 s, 55°C for 30 s (annealing), 72°C for 30 s (extension) and a final 72°C for 5 min. Amplicons were cleaned with the AMPure beads and used as template in a second PCR (50 µL reactions) for 8 cycles to attach dual indices and sequencing adapters. The amplicons were checked for size and purity in 2% agarose gels (Figure S1, Supporting Information). Amplicons (excluding Ae23 which was too low in DNA concentration) were adjusted to the same 4 nM concentration, pooled, denatured and paired-end sequenced in house following the Nextera protocol (Illumina Inc). For genome sequencing, a total of 680 ng Ae24 DNA (20 µL) was delivered to the Norwegian sequencing centre in Oslo. The DNA was run through a SPRIworks protocol (Beckmann) using a size selection of 300–600 bp and 10 PCR cycles.

### Sequence analysis of rRNA genes

The ribosomal 16S rRNA gene sequences were analysed in mothur (Schloss *et al*. 2009). Following the MiSeq standard operating procedure, fastq files were merged from paired reads and filtered to remove adapters, primers and sequences with ambiguous nucleotides, homopolymers (>7 nt), short length (<400 nt) and potential chimeras found relative to the more abundant sequences using VSEARCH (Rogers *et al*. 2016). The sequences were aligned in SILVA 138 (Quast *et al*. 2013) and screened to overlap in the same V3–V4 region. Sequence numbers were normalized across samples by subsampling to the smallest sample. Operational taxonomic units (OTUs) were clustered at 97% similarity to define species and singletons were removed. Classification was performed with the SILVA 138 database at a cutoff of 80 and inferred to the lowest possible taxonomic level, using the method implemented by RDP (Wang *et al*. 2007). Sequences not classified as Bacteria and Archaea at the domain level (Eukaryota, chloroplast, mitochondria, unknown) were removed. The resulting taxonomy was supported by searching GenBank (blastn) for nearest relatives, identities and sample source information (Altschul *et al*. 1997).

Shotgun MiSeq files (fastq) were filtered for adapters and quality trimmed with default parameters using the IDBA_UD implemented Sickle (Peng *et al*. 2012). These rRNA gene sequences were also classified using mothur (Schloss *et al*. 2009). Following the merging of paired reads and filtering to remove sequences with ambiguous nucleotides and homopolymers, classification was performed at a cutoff of 100. Taxa still unidentified following searches of random sequences by blastn, were classified at a cutoff of 80 and again searched by blastn. Remaining unknowns were searched (blastn) against the phylotypes of ‘*Ca*. A. aggregatus’ (EF508132, GQ240891, GQ240892 and HQ412802- HQ412805) and *A. excavata* (GQ240893 and KX713266).

### Sequence assembly, binning and functional gene analysis

For the genome assembly, reads were merged using Fq2fa and assembled in IDBA_UD (Peng *et al*. 2012). All contigs above 1 kb in length were uploaded to the Integrated Microbial Genomes (IMG) Expert Review for functional annotation (Markowitz *et al*. 2014). Functions were represented by clusters of orthologous groups (COGs) and if missing, alternatively by protein families (pfam) or the KEGG orthology (KO) used to analyse pathways. The carbohydrate active enzymes were represented by annotations from dbCAN2 (Zhang *et al*. 2018) for improved detection. Secreted proteins were predicted using LipoP 1.0 (Junker *et al*. 2003) and SignalP 5.0 (Armenteros *et al*. 2019).

The ‘*Ca*. A. aggregatus’ genome was binned from the full >1 kb data set based on sequence composition (k-mer) for taxonomic classification using PhyloPythiaS (Patil, Roune and McHardy 2012). Composition-based binning is quite accurate for low diversity microbiomes dominated by a single identified target population and has been used to reconstruct genomes from a biomass digester (Hagen *et al*. 2017). The classifier was trained with contigs similar to ‘*Ca*. A. aggregatus’ and potential contaminants (Supplementary Text). Genome completeness and contamination was assessed with 507 marker genes from 263 gammaproteobacterial genomes using CheckM (Parks *et al*. 2015).

A phylogenomic analysis was performed with reference genomes guided by the previous 16S rRNA gene phylogeny (Jensen *et al*. 2010). For each genome, 37 single copy marker genes (Darling *et al*. 2014) expanded from AMPHORA (Wu and Eisen 2008) were identified and aligned in Phylosift. Multiple hits (e.g. IF-2) were manually inspected and only sequences of the most significant blastp hits were retained. Less complete genomes without the full set of markers genes (e.g. *Phacoides* symbiont) were not retained for phylogenomic analysis. The markers were concatenated into a single alignment using BioEdit (Hall 1999). The carbohydrate-active lytic polysaccharide monooxygenase (LPMO10) gene deduced amino acids were aligned in Muscle (Edgar 2004). All phylogenetic analyses were performed in Phylip (Felsenstein 2013).

To quantitatively compare functional gene abundances in ‘*Ca*. A aggregatus’ and reference genomes, mothur (Schloss *et al*. 2009) was used to generate a normalized and nonredundant dataset on the COGs by subsampling the genes ‘shared file’ (COG table). Singletons were removed and differential gene abundance in COGs between Endozoicomonadaceae and other Gammaproteobacteria was assessed using a linear discriminant analysis (LDA) effect size (LEfSe) method that couples standard Kruskal–Wallis and Wilcoxon tests with tests encoding biological consistency and effect relevance (Segata *et al*. 2011), as implemented in mothur (Schloss *et al*. 2009). For gene abundance differences between ‘*Ca*. A. aggregatus’ and other Endozoicomonadaceae, a miniumum 2-fold increase in COG relative abundances was adopted as a threshold (Karimi *et al*. 2018). The COG table was also used to calculate a distance matrix (Bray–Curtis) for principal coordinate analysis (PCoA). Selected COGs responsible for shifting samples along the two axes were included in the PCoA visualization. The clustering was judged for statistical significance (*P* < 0.05) using the nonparametric group test of variance (AMOVA).

### Protein extraction

To support the metagenome with expressed genes, thus organism activity, proteins were identified. Proteins were extracted from four clams by combining tissue with liquid phenol (5 g in 0.5 mL H_2_O), 0.4 M sucrose and glass beads (size ≤ 106 µm) and bead beating in a FastPrep24 (MP Biomedicals, Santa Ana, CA) for 3 × 60 s. Debris was removed by centrifugation at 16 000 × *g*, and the top phase was transferred to a new tube and combined with an equal volume of 1 M sucrose. After vortexing and re-centrifugation, the top phase was transferred to a new tube and five volumes of 0.1 M ammonium acetate in methanol were added. Samples were incubated at −20°C overnight and the next day centrifuged at 16 000 × *g* for 20 min. The pellet was washed with cold acetone and solubilized in SDS sample buffer. Samples were loaded on an Any-kD Mini-PROTEAN gel (Bio-Rad Laboratories), separated by a 5-minute electrophoresis run (minor separation, but mostly to clean up sample) and stained using Coomassie Brilliant Blue R250. Proteins entrapped in the gel were reduced and carbamidomethylated using 10 mM DTT and 55 mM iodacetamide, respectively, prior to in-gel digestion with trypsin as described previously (Arntzen *et al*. 2015).

### Mass spectrometry

Prior to mass spectrometry, peptides were desalted using C18 ZipTips (Merck Millipore, Darmstadt, Germany), according to manufacturer's instructions. Peptides were analyzed using a nanoLC-MS/MS system (Dionex Ultimate 3000 UHPLC; Thermo Scientific, Bremen, Germany) connected to a Q-Exactive mass spectrometer (Thermo Scientific) and operated in data-dependent mode to switch automatically between orbitrap-MS and higher-energy collisional dissociation (HCD) orbitrap-MS/MS acquisition. MS raw files were analyzed using MaxQuant (Cox and Mann 2008) version 1.6.0.13 and searched against all predicted proteins from the ‘*Ca*. A. aggregatus’ genome (1772 contigs) in the background of the whole metagenome (128 012 contigs). This adds confidence to the 577 detected proteins by ensuring that any identified peptide was supported by an underlying gene. MaxQuant parameters were as previously described (Hagen *et al*. 2017). Identifications were filtered to require a minimum of two positives for detection (singletons removed) and to achieve a protein false discovery rate (FDR) of 1%.

### Data deposition

Metagenome amplicon and shotgun data sets are available at the NCBI Sequence Read Archive under accession number PRJNA523603. Contig data sets are available at the JGI system Integrated Microbial Genomes under accession number Ga0072491. The mass spectrometry proteomics data sets are available at the ProteomeXchange Consortium via the PRIDE partner repository with the identifier PXD016288.

## RESULTS AND DISCUSSION

### A distinct gill microbiome

Phase contrast microscopy and DNA fragment screening of gill samples from the *A. excavata* clams indicated that representatives of ‘*Ca*. A. aggregatus’ were present. The observed structures resembled aggregations scattered inside vacuoles as previously seen by fluorescent *in situ* hybridization and the restriction enzyme digestion profiles matched a previously cloned 16S rRNA gene sequence (Figure S1, Supporting Information; Jensen *et al*. 2010). We estimated a total of 10^6^ aggregates and 10^8^ ‘*Ca*. A. aggregatus’ cells per clam gill microbiome.

**Figure 1.**
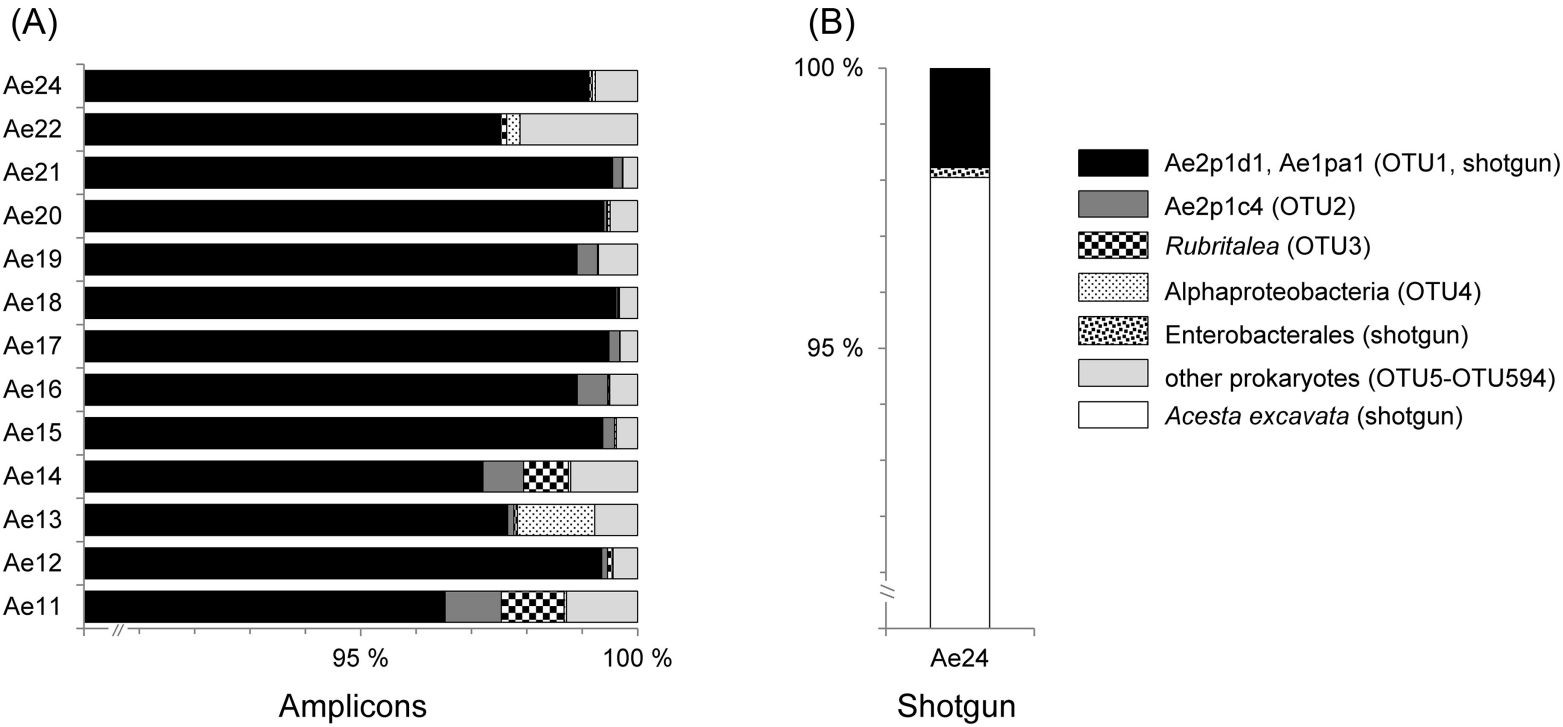
Microbiome diversity associated with gills of *Acesta excavata* clams from deep-water coral reefs off mid-Norway. **(A)** Bacteria and Archaea represented by V3–V4 regions simultaneously amplified from 16S rRNA genes totalling 39 500 sequences per clam. **(B)** Shotgun sequenced gill microbiome of clam Ae24 totalling 3994 identified 16S rRNA and 18S rRNA gene fragments. The clams were collected from reefs in Skarv (369 m depth; 65° 40′ N, 07° 32′ E) and Kristin (317 m depth; 65° 01′ N, 06° 32′ E) hydrocarbon fields at Haltenbanken.

The V3–V4 region of the 16S rRNA gene was PCR amplified from Bacteria and Archaea (Takahashi *et al*. 2014) from gill tissue samples of 13 clams. In total 39 500 normalized sequence reads were analysed per gill microbiome. The OTUs estimated a high Good's coverage (>99%) and a low Shannon's H (<0.24) but the rarefaction curves did not reach an asymptote (Figure S2, Supporting Information). This probably resulted from a greatly enriched OTU1 (Fig. 1A). OTU1 (>95% read abundance) exactly matched phylotypes Ae2p1d1 and Ae1pa1 that were previously found dominating gill tissue clone libraries from *A. excavata* (Jensen *et al*. 2010). Another phylotype (Ae2p1c4) from the libraries was 99% identical to OTU2 (Fig. 1A). The slightly different 16S rRNA genes within the libraries is similar to aggregates of tropical coral associated *Endozoicomonas* (<3% nucleotide variation), indicating different co-occurring strains (Neave *et al*. 2016) or a few to one dominant strain with different gene sequences (Neave *et al*. 2014; Ding *et al*. 2016). The ‘*Ca* A. aggregatus’ phylotypes are most similar (∼94% identity) to the *Kistimonas*-like gill symbiont of the *Phacoides* clam (Lim *et al*. 2019). Our study confirms that *A. excavata* enriches for distinct uncultured bacteria, in line with the conserved bacterial diversity patterns observed in corals on deep-water reefs (Jensen *et al*. 2019). The remaining 591 OTUs (<5% read abundance) included 19 prokaryotic phyla (Fig. 1A). For example, OTU3 affiliated 99% with a sponge hosted *Rubritalea* (Verrucomicrobia; EU346428). Archaea were represented by only 4 OTUs and classified as Nitrososphaeria (Crenarchaeota) and Woesearchaeales (Nanoarchaeota).

The amplicon sequencing was supported by the shotgun sequencing. Among the 3994 shotgun rRNA gene sequences identified (clam Ae24), 16S rRNA gene sequences were dominated by ‘*Ca*. A. aggregatus’ phylotype Ae2p1d1 (Fig. 1B). The 18S rRNA gene sequences all identified as *A. excavata* (Fig. 1B). This suggested much remaining eukaryal DNA following bacterial DNA enrichment (Figure S1, Supporting Information). Some protists and fungi might have escaped detection because they may not have been represented in the database or because a too stringent classification was employed. To include this uncertainty, the term gill microbiome is used.

### A dominant, deep branching Endozoicomonadaceae

**Figure 2.**
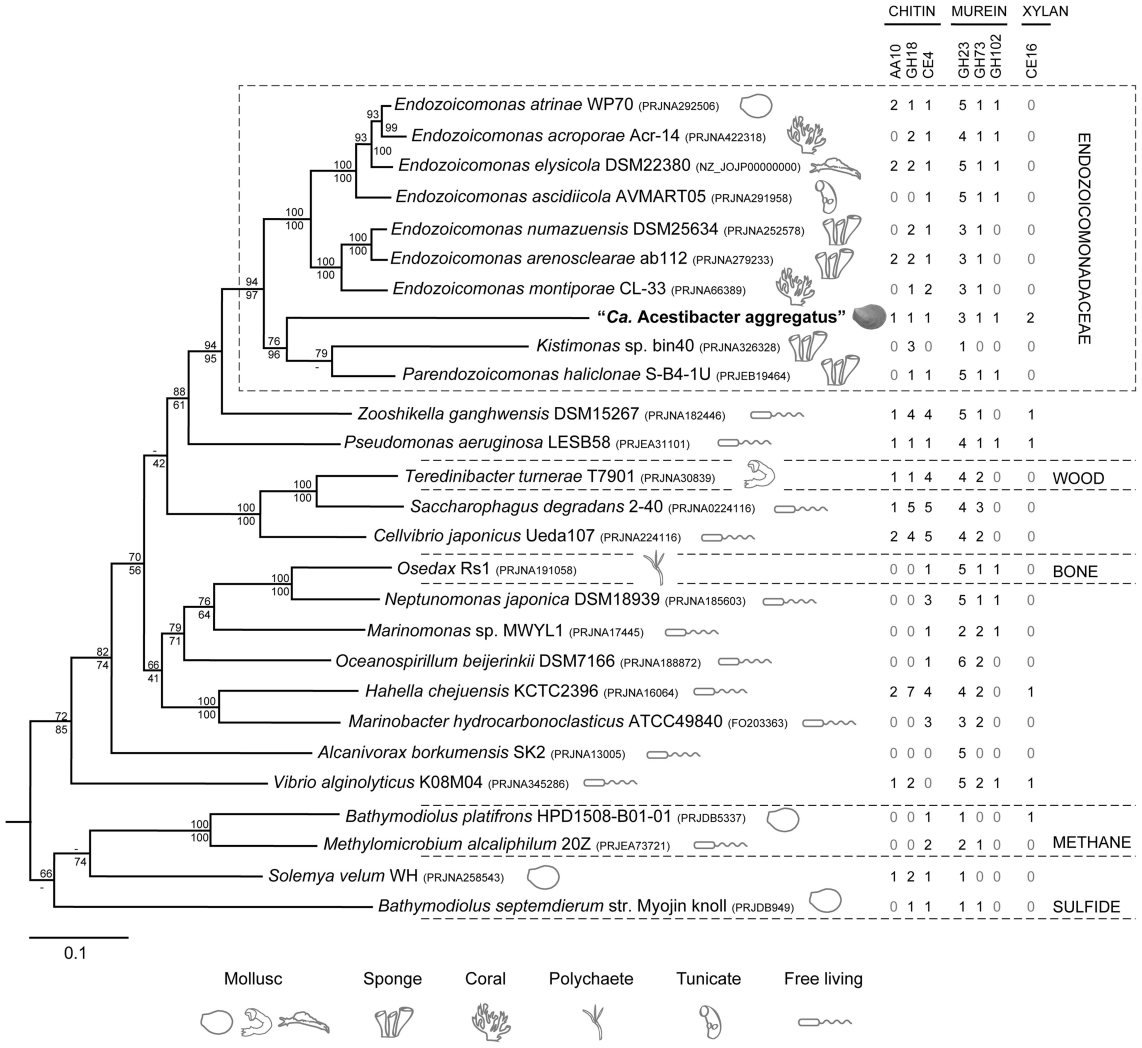
Phylogenomic tree showing the relationship of ‘*Candidatus* Acestibater aggregatus’ to 26 gammaproteobacterial references. Profiled across taxa are gene counts for depolymerizing enzymes, predicted secreted and active on structural carbohydrates in exoskeletons of zooplankton (chitin) and cell walls of bacteria (murein i.e. peptidoglycan) and algae (xylan). Included in the tree are marine nutritional symbionts from the host families Teredinidae, Siboglinidae, Solemyidae and Mytilidae (wood, bone, sulfide and methane utilizers). The tree is based on a concatenated alignment of 5950 amino acids deduced from 37 single copy genes (Darling *et al*. 2014) and was constructed in Phylip (Felsenstein 2013) using maximum likelihood (PAM model). Bootstrap (SEQBOOT) values above 50% from 100 iterations (CONSENSE) are indicated at the branch points (PROML/PROTPARS). The scale bar represent 0.1 changes per amino acid position. The outgroup was the Alphaproteobacterium *Agrobacterium tumefaciens* Ach5 hosted by a sneezewort (PRJNA278497).

Using a 16.2 kb contig in a training set for taxonomic classification by PhyloPythiaS (Patil, Roune McHardy 2012), a 4.5 Mb ‘*Ca*. A. aggregatus’ genome was drafted (Table 1). A complete rRNA operon was present and shared 99% identity to phylotype Ae2p1d1 (16S), 88% to a virulent *Pseudomonas aeruginosa* from a burn patient blood culture (23S) and 92% to a *Halioglobus japonicus* from seawater at 100 m depth at the Pacific Station S1 (5S). The 16S rRNA gene sequence differed from that of Ae2p1d1 by only one nucleotide indicating assembly accuracy. The difference (C) was found in position 1511 (*Escherichia coli* numbering) of primer 1492r used in PCR prior to the previous cloning. CheckM (Parks *et al*. 2015) estimated genome completeness at 83.0% with a contamination of 1.3% (Table 1). This lack of completeness is likely caused by genes associated with unbridged repeats (Ding *et al*. 2016), misplaced contigs (inaccurate binning) and remaining host DNA (insufficient enrichment). As the genome is not closed, undetected genes cannot be excluded. Ideally, assembly would have been performed using DNA from a cultured ‘*Ca*. A. aggregatus’ but attempts at culturing were not successful. However, because shotgun methods sample almost randomly, it is likely that the most abundant genes and proteins were detected. The genome was searched for 37 ‘elite’ marker genes previously identified as near universal, in single copy and individually reconstructing similar phylogenetic trees (Darling *et al*. 2014) and all were identified. Obtained phylogeny is congruent with a previous single gene 16S rRNA based phylogeny (Jensen *et al*. 2010) and positioned the ‘*Ca*. A. aggregatus’ in a deep branching lineage adjacent to temperate sponge-hosted *Kistimonas* bin40 (Slaby *et al*. 2017) and *Parendozoicomonas* (Bartz *et al*. 2018) in a clade of marine invertebrate associated Oceanospirillales (Fig. 2; Bayer *et al*. 2013; Neave *et al*. 2016, 2017; Slaby *et al*. 2017; Lim *et al*. 2019; Robbins *et al*. 2019), family Endozoicomonadaceae.

**Table 1.** Genome features of ‘*Candidatus* Acestibacter aggregatus’.

	Ae24
Mbp, %GC	4.5, 35
Coverage	31 × (1830 contigs)
Completeness	83% (1.3% contamination)
Genes	5611 (1872 functional)
Proteins	12 (577 including host)
COGs	1711
rRNA	16S (1), 23S (1), 5S (2)
tRNA	148
CRISPR	Cas3 (1)
Sequences total	∼19 million (∼310 bp)

### Genomic evidence for host–symbiont integration

**Figure 3.**
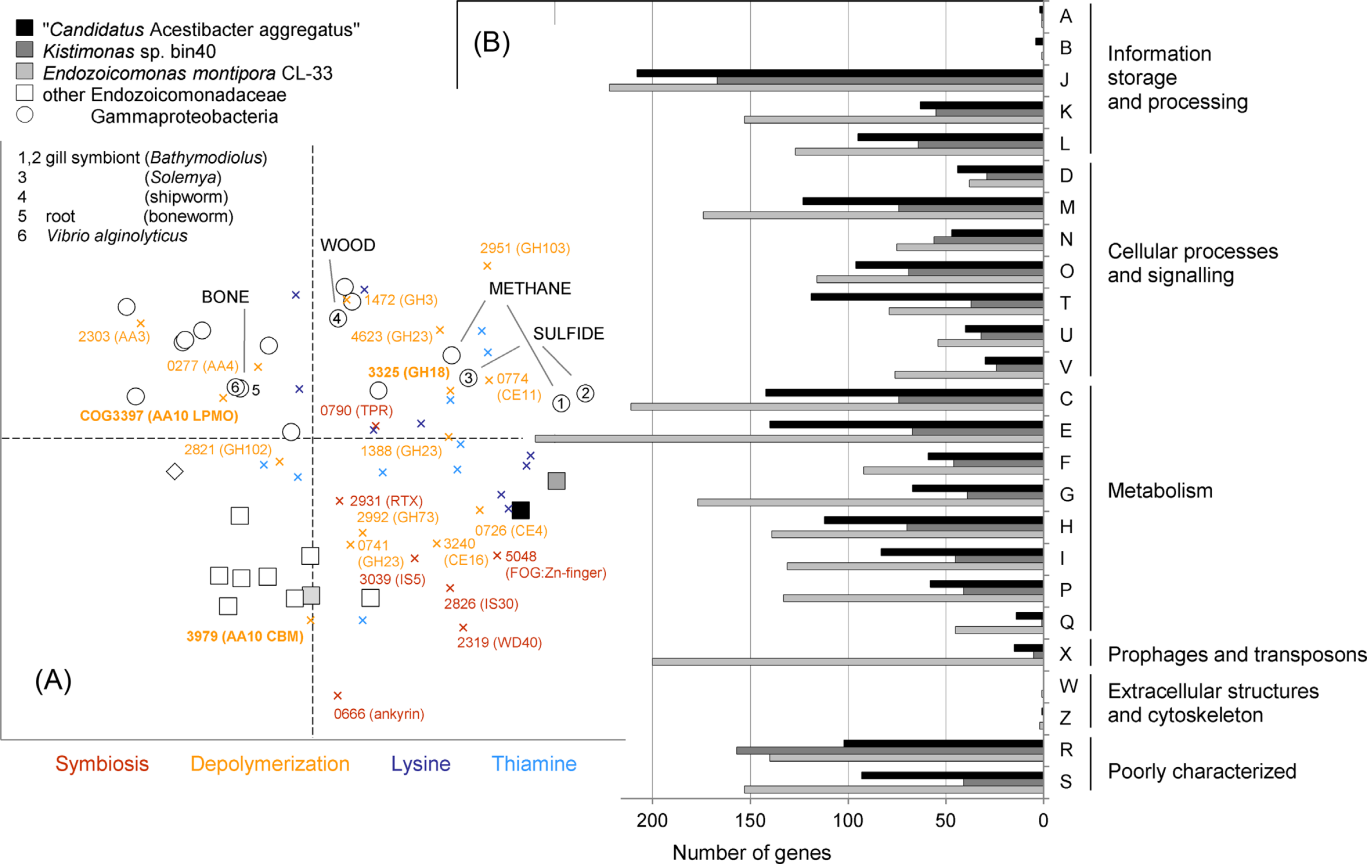
Functional comparison summarizing variation in gene counts across COGs for ‘*Candidatus* Acestibacter aggregatus’ and 27 gammaproteobacterial references. **(A)** Gene counts per COG principal coordinate analysis in comparison with all references and indicates functions potentially attributable to symbiosis, depolymerization and nutrition. COGs in bold are hypothesized to represent a minimal chitinolytic machinery of secreted AA10 and GH18 enzymes. The round and squared shapes represent individual genomes. The crosses represent individual COGs, for clarity named for those not part of a pathway only. **(B)** Gene counts per COG category in comparison with a phylogenetically close (bin40) and a more distant (CL-33) Endozoicomonadaceae. Each genome is represented by 1107 genes and gene counts are compared for a total of 2631 COGs. PCoA axes range −0.35 to 0.35 and explain a variation of 10.9% (*x*-axis) and 8.3% (*y*-axis). The genome of the outgroup is represented by a diamond (Ach5).

To investigate the functional characteristics and potential benefits to the host, inferred amino acid sequences were compared across the genomes of ‘*Ca* A. aggregatus’ and 27 Gammaproteobacteria references (Fig. 2) using COGs. All genomes were normalized to the same number of COGs (2631) and annotated using the same COG database (IMG). Clustering the COGs by PCoA (Fig. 3A) separated the Endozoicomonadaceae from the other Gammaproteobacteria (AMOVA *P* < 0.001), supporting homogeneity within the family and highlighting differences with other Gammaproteobacteria (Fig. 2, bootstrap >93%). Comparable to the marker genes (Fig. 2), COGs clustered ‘*Ca*. A. aggregatus’ with the sponge-hosted *Kistimonas* bin40 (Fig. 3A). The COG profile resembled *Kistimonas* bin40 (Slaby *et al*. 2017) and the more distant coral-associated *E. montipora* CL-33 (Ding *et al*. 2016; Fig. 3B).

A set of 66 COGs were found significantly enriched in Endozoicomonadaceae compared to other Gammaproteobacteria (Figure S3, Supporting Information; Supplementary Text). A total of 50 of these COGs were represented by the ‘*Ca*. A. aggregatus’ partial genome. The set was enriched in repeats, hemolysins, adhesins and secretion systems, but depleted in chemotaxis and motility genes. This apparently reflects host association comparable to proteobacterial symbionts including Endozoicomonadaceae in sponges (Slaby *et al*. 2017; Karimi *et al*. 2018), corals (Neave *et al*. 2017; Robbins *et al*. 2019) and the *Phacoides* clams (Lim *et al*. 2019). The most enriched COGs were ankyrin repeats (Figure S3, Supporting Information). Ankyrins (COG category T) are eukaryote-like proteins that can subvert host defences and protect against leucocytes and other host immune cells (Al-Khodor *et al*. 2010). Repeats may also flank mobile elements like the highly enriched transposons (COG category X) of the *E. montipora* CL-33 genome (Fig. 3B). The enrichment was suggested to be a signature of ongoing genome erosion associated with a symbiotic or a pathogenic lifestyle (Ding *et al*. 2016). COGs of repeats (ankyrins, TPR, RTX) and transposons (IS5, IS30) in ‘*Ca*. A. aggregatus’ were found >2-fold enriched above the average Endozoicomonadaceae (Supplementary Text). RTX (repeat in toxins) is a family of secreted cytolytic toxins of activities like pore forming hemolysins, colonization and virulence (Linhartová *et al*. 2010). RTX of the sulfur oxidizing gill symbiont of *Bathymodiolus* have been hypothesized to be ‘tamed’ for beneficial interaction by potentially being antagonistic towards parasitic bacteria (Sayavedra *et al*. 2015). As the *Bathymodiolus* sulfur oxidizer might be evolving into an obligate symbiont, its many expressed chaperones, COG category O (Fig. 3B), and histone-like proteins may protect against protein misfolding from enhanced mutation (Ponnudurai *et al*. 2017). The proteome of ‘*Ca*. A. aggregatus’ also revealed activities (Table 2) of chaperones and notably amino acid synthesis (elongation factor), energy production (ATP synthase) and polysaccharide degradation (LPMO10). Support for host-symbiont integration was provided by the many potential symbiosis factors identified (Table S1, Supporting Information).

**Table 2.** Proteins detected from ‘*Candidatus* Acestibacter aggregatus’. Triplicate samples of gills and guts plus a gut sample of clam Ae24 was analysed. A total of four proteins were hypothetical (10000617^1^, 10001394, 10002442^1^ and 10006042^1^).

				Aligned	Positive clams		
Gene ID	COG	Function	Closest relative (accession no.)	(ID %)	G	g	g
100002212	0443	Chaperone DnaK	*Kistimonas* gill symbiont (OQX39667)^M^	648 (84)	3	3	1
10006401	0056	F0F1 ATP synthase	*Parendozoicomonas h*. (WP087109735)^S^	514 (78)	3	3	1
10003315	0050	Elongation factor Tu	Cellvibrionaceae sp. (WP142929796)^Fr^	397 (90)	3	0	0
100000514	00816^2^	DNA binding H-NS	*Kistimonas* gill symbiont (OQX36710)^M^	139 (65)	3	0	0
10002248^1^	2885	Membrane OmpA	*Kistimonas* gill symbiont (OQX38305)^M^	169 (57)	3	0	0
10001183^1^	1651	Periplasmic DsbC	*Zooshikella ganghwe*. (WP094788118)^Fr^	223 (44)	3	0	0
100000713^1^	3397	Enzyme LPMO10	*Vibrio alginolyticus* (ARP38077)^F^	501 (76)	2	0	1
10001996^1^	3637	Membrane Porin	*Oleispira antarctica* (WP046008914)^Fr^	187 (40)	1	2	0

G Gill (*n* = 3), g gastrointestinal tract (*n* = 3 + 1), ^M^mollusc, ^S^sponge, ^F^fish, ^Fr^free-living, ^1^signal peptide, ^2^pfam.

### Biosynthesis pathways suggestive of nutritional symbiosis

The relationship with ‘*Ca*. A. aggregatus’ may provide essential missing nutrients due to the presence of genes encoding the production of amino acids and B vitamins. COG category E ‘amino acid transport and metabolism’ represents 140 genes in ‘*Ca*. A. aggregatus’ (Fig. 3B). The elongation factor was expressed (Table 2) and genes for all 20 tRNAs and 18 aminoacyl-tRNA synthetases (Gottschalk 1988) were found. Complete pathways were identified for the synthesis of 11 amino acids including the essential threonine, phenylalanine and tryptophan. Remaining synthesis pathways were missing some genes, like *dapE* for lysine and *hisN* for histidine (Figure S4, Supporting Information). Curiously, *hisN* was neither found in the Endozoicomonadaceae reference genomes (Fig. 2). Apart from building proteins, amino acids are precursors for the synthesis of such compounds as purines and pyrimidines and many B vitamins required as cofactors of enzymes in the metabolism of, for example, NAD (Gottschalk 1988). The synthesis of B vitamins belongs to COG category H ʻcoenzyme transport and metabolismʼ, representing 112 genes in ‘*Ca*. A. aggregatus’ (Fig. 3B). Complete pathways were identified for the synthesis of thiamine (B1), riboflavin (B2), nicotinate (B3), panthotenate (B5), pyridoxine (B6) and biotin (B8) with folate (B9) missing the gene *phoAB* (Figure S4, Supporting Information). Amino acids and co-factors are typically produced by nutritional symbionts as were noted for the sulfur- and methane-oxidizing gill symbionts of *Bathymodiolus* (Ponnudurai *et al*. 2017; Supplementary Text). Genes for nitrogen fixation (*nif*) were not found but the nutritional syntheses may recycle nitrogen by assimilating host-excreted ammonia via glutamate dehydrogenase or the GOGAT glutamine and glutamate synthetase (Gottschalk 1988; Table S2, Supporting Information).

### Heterotrophic central metabolism

The Endozoicomonadaceae are heterotrophs utilizing carbohydrates and proteins (Neave *et al*. 2016, 2017; Bartz *et al*. 2018). Genes encoding CO_2_ assimilation, oxidation of sulfur or methane was not found in ‘*Ca*. A. aggregatus’ (Supplementary Text). Energy appeared to be generated by electrons carried from the oxidation of NADH. Identified genes revealed a respiratory chain with several types of cytochromes including a terminal oxidase cbb3 with high affinity for O_2_ common to organisms in low oxygen environments (Table S2, Supporting Information). Hydrogen peroxide and radicals formed from the reaction of oxygen with reduced flavoproteins or other electron carriers (Gottschalk 1988) may be protected against by the identified catalase and superoxide dismutase encoding genes.

NADH and many precursors for biosynthesis are produced by the TCA cycle, of which all genes were found (Table S2, Supporting Information). TCA may also operate on acetate (Gottschalk 1988) by the glyoxylate cycle enzymes isocitrate lyase and malate synthase but whether acetate is used by ‘*Ca*. A. aggregatus’ is unclear. The gene for acetyl-CoA synthetase (EC:6.2.1.1) was not found. TCA may however be fuelled by Acetyl-CoA from fatty acid beta oxidation encoded by genes for fatty acid import (*fadL, D*) and degradation (*fadA, B, E*and*H*; Table S2, Supporting Information; Supplementary Text). Acetyl-CoA may also be formed by the degradation of peptides such as alanine or cysteine. The NADPH and five carbon sugars for nucleotide and NAD synthesis are produced by the pentose phosphate cycle (Gottschalk 1988), but genes encoding NADP^+^ reduction (EC:1.1.1.44 and EC:1.1.1.49) were not found (Table S2, Supporting Information), not even in the Endozoicomonadaceae reference genomes (Fig. 2). The ‘*Ca*. A. aggregatus’ genome was also missing genes encoding the glycolysis enzymes (Table S2, Supporting Information) glucose phosphate isomerase (EC:5.3.1.9) and pyruvate kinase (EC:2.7.1.40). The kinase could be replaced by a pyruvate phosphate dikinase (ppsA). The glucose could be replaced by fructose-6P from chitin, which also yields acetate and ammonia (Keyhani and Roseman 1999). Genes encoding all five missing enzymes (EC denoted) were, however, found in the *A. excavata* host genome. From chitin, the pathway was indicated complete for peptidoglycan biosynthesis, but genes were missing between the monomer GlcNAc (N-Acetylglucosamine) and fructose-6P. Furthermore, the gene for the glucose phosphotransferase import system enzyme I (Gottschalk 1988) was also missing (EC:2.7.3.9). Thus, it is unclear whether ‘*Ca*. A. aggregatus’ can utilize glucose.

Glucose is mostly sourced from photosynthetates of algae (Bergauer *et al*. 2018) and because it is preferentially used by bacteria (Gottschalk 1988) and other microplankton, sustenance in the deep-water coral reef environment may instead involve recalcitrant compounds that are more abundant. The ‘*Ca*. A. aggregatus’ genome was found to encode several enzymes that may depolymerize carbohydrates (26), proteins (40), peptides (32) and lipids (10) (Table 3 and Table S3, Supporting Information). Many of these enzymes were identified with signal peptides suggesting transport out of the cell.

**Table 3.** Depolymerizing enzyme profiles targeting marine structural polysaccharides in ‘*Candidatus* Acestibacter aggregatus’. Gene count is shown in comparison with genomes from 27 gammaproteobacterial references.

				Aligned	No. of genes		
Gene ID	CAZy	Function	Closest relative (accession no.)	(ID %)	A	E	O
100003712	AA3	GMC-oxidoreductase	*Kistimonas* gill symbiont (OQX35095)^M^	483 (63)	1	18	30
10000374	AA4	FAD-oxidoreductase	*Zooshikella ganghwe*. (WP027709861)^Fr^	461 (59)	1	2	17
100000713^1^	AA10	Monoooxygenase	*Vibrio alginolyticus* (ARP38077)^F^	501 (76)	1	6	9
10001412^1^	CE4	Chitin deacetylase	*Kistimonas* gill symbiont (OQX33362)^M^	329 (47)	1	10	37
10000605	CE11	Amine deacetylase	(OQX37986)^M^	304 (88)	1	10	17
10001024^1^	CE16	Acetyl esterase	(OQX34880)^M^	303 (42)	2	1	5
10001957	GH3	Acetylhexosaminidase	(OQX35778)^M^	332 (55)	1	16	34
10000872^1^	GH18	Chitinase	Sulfur-oxid. gill symb. (WP066043100)^M^	544 (48)	1	14	25
10000305^1^	GH23	Peptidoglycan lytic	*Kistimonas* gill symbiont (OQX38807)^M^	518 (57)	3	36	77
10000014^1^	GH73	Peptidoglycan hydrol.	(OQX39118) ^M^	218 (44)	1	9	23
10000317^1^	GH102	Lysozyme	(OQX37468)^M^	372 (57)	1	6	5
10004641	GH103	Peptidoglycan lytic	*Parendozoicomonas h*. (WP087107845)^S^	296 (56)	1	10	25

A ‘*Ca*. A. aggregatus’, E Other Endozoicomonadaceae (*n* = 10), O Other Gammaproteobacteria (*n* = 17), ^M^mollusc, ^S^sponge, ^F^fish, ^Fr^free-living, ^1^signal peptide.

### Potential for complex polysaccharide degradation

Outside the cell, secreted enzymes may degrade refractory carbon compounds available in marine snow and resuspended matter (Schlüter *et al*. 2000) as well as fresher biomass from down-welling (Thiem *et al*. 2006). Springtime plankton detected above Haltenbanken reefs included, for example, metabolically-active *Microcalanus* copepods, *Phaeocystis* and *Pyramimonas* algae, *Thalassiosira* diatoms, *Gyrodinium* dinoflagellates and SAR11 bacteria (Jensen *et al*. 2015). This provides nutrition from the more recalcitrant and structurally similar compounds of bacterial cell wall peptidoglycan, algae cell wall cellulose and xylan and the zooplankton exoskeleton chitin. Chitin is considered the most abundant marine polysaccharide, and it is continuously supplied as a result of zooplankton molting (Keyhani and Roseman 1999).

**Figure 4.**
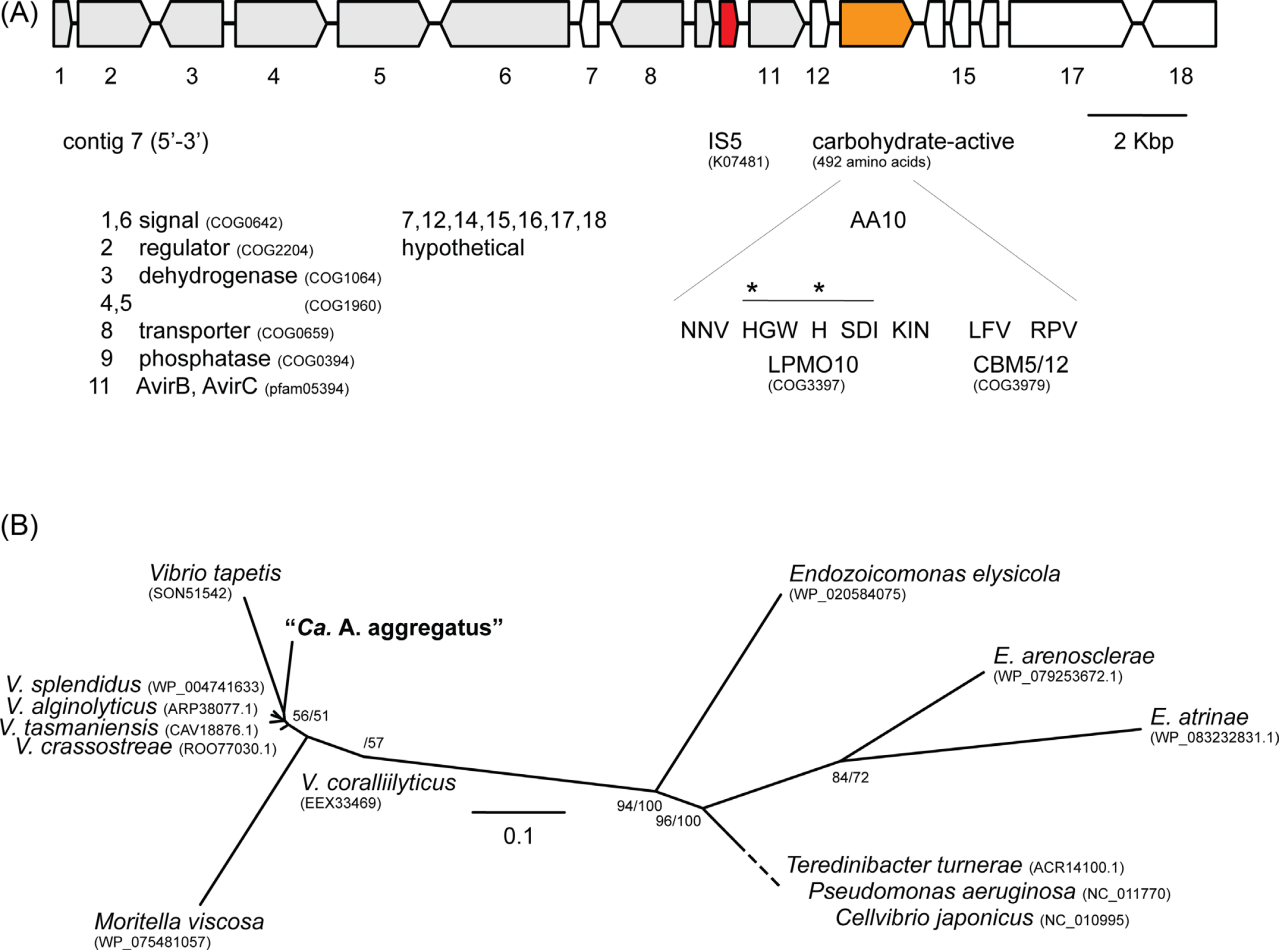
Neighborhood analysis of the carbohydrate-active domain containing lytic polysaccharide monooxygenase in ‘*Candidatus* Acestibacter aggregtus’. **(A)** Gene annotation is shown highlighting amino acid residues flanking the family auxiliary activity 10 (AA10) LPMO catalytic domain and the chitin binding CBM5/12 module. Residues bridged by a line flank the alignment. Asterisks (*) indicate Cu coordinating histidines (H28 and H114) conserved in the active site. **(B)** Phylogenetic tree showing the relationship of the ‘*Ca*. A. aggregatus’ LPMO10 to three other Endozoicomonadaceae and ten other Gammaproteobacteria as based on the 220 aligned (Edgar 2004) catalytic domain amino acids. The tree was constructed in Phylip (Felsenstein 2013) using maximum likelihood (PAM model). Bootstrap (SEQBOOT) values above 50% from 100 iterations (CONSENSE) are indicated at the branch points (PROML/PROTPARS). The scale bar represent 0.1 changes per amino acid position. No outgroup was included because of the well separated clustering.

Interestingly, the gene and protein of a copper dependent monooxygenase were identified (Tables 2 and 3). This auxiliary activity 10 (AA10) family lytic polysaccharide monooxygenase (LPMO) contains a C-terminal family 5 chitin binding module CBM5/12 indicating activity towards chitin (Fig. 4A). The conserved amino acid residues, including the two Cu coordinating histidines (H28, H114) of the active site and the surrounding alanine (A112) and phenylalanine (P187), resemble characterized LPMO (CBP21) from the chitin-utilizing *Serratia marcescens* (Vaaje-Kolstad *et al*. 2010; Book *et al*. 2014). Active LPMOs are known to cleave glycosidic bonds in recalcitrant polysaccharides by oxidation, increasing the accessibility for hydrolases and esterases, thus boosting the enzymatic degradation of chitin and cellulose (Vaaje-Kolstad *et al*. 2010; Hemsworth *et al*. 2015).

LPMO homologues have been identified in other symbiotic bacteria, such as *Teredinibacter turnerae* (Sabbadin *et al*. 2018). It is known that in gills of this bivalve host (shipworm), *T. turnerae* secrete enzymes that help efficiently degrade cellulose (Distel, DeLong and Waterbury 1991; Yang *et al*. 2009; OʼConnor *et al*. 2014). Surprisingly, the closest homologous sequence (76% amino acid identity) of the ‘*Ca*. A. aggregatus’ LPMO is from a *Vibrio*, associated with a Kiel fjord pipefish (Table 3). Inside hosts, LPMOs may become active factors in the adhesion of symbionts and virulence of pathogens (Agostoni, Hangasky and Marletta 2017). Whether LPMOs in gill associated *Endozoiciomonadaceae* are involved with parasitism on chromatin in *Bathymodiolus* (Zielinski *et al*. 2009) or somehow with pathogenicity of razor clams (Elston 1986), blue mussels (Schill, Iwanowicz and Adams 2017), king scallops (Cano *et al*. 2018) or fish larvae (Mendoza *et al*. 2013; Katharios *et al*. 2015) remains unknown. Phylogenetic analysis positioned the ‘*Ca*. A. aggregatus’ LPMO in a clade with another coral reef derived LPMO in the tree (Fig. 4B). All the ‘*Ca*. A. aggregatus’ clade members associate with potentially pathogenic Vibrionaceae and Moritellaceae. These bacteria encode uncharacterized LPMO enzymes that fall outside the two phylogenetic clades of Book *et al*. (2014). Clade I contains all biochemically defined chitin monooxygenases, while clade II contains subclades that are either cellulose or chitin monooxygenases. In the Fig. 4B alignment, the ‘*Ca*. A. aggregatus’ and its seven clade members share the same 185 amino acid residue length but only 36 of the 118 conserved residues are shared with the aforementioned CBP21 from *Serratia*. The CBP21 is 19 residues shorter and clusters in clade I (Book *et al*. 2014). Although these LPMOs are indicated with a chitin binding module CBM5/12, chitin may not be the catalytic domain's only substrate (Supplementary text). Genes for chitin metabolism in ‘*Ca*. A. aggregatus’ may have been acquired by horizontal transfer from the Vibrionaceae, as indicated from the LPMO phylogeny, elevated 43 mole % GC and nearby IS5 element (Fig. 4A).

The LPMO of ‘*Ca*. A. aggregatus’ may form a minimal chitinolytic machinery together with a CBM5/12 chitin-binding GH18 glycosyl hydrolase as found in S. *marcescens* (Vaaje-Kolstad *et al*. 2013; Table 3). The combined oxidative and hydrolytic activity may strengthen evidence for complex polysaccharide degradation. Chitin may also be deacetylated by a CE4 carbohydrate esterase. These and other polysaccharide active enzymes contain signal peptides for secretion (Table 3) and are likely exported out of the ‘*Ca*. A. aggregatus’ by type 2 secretion systems T2SSs (Table S1, Supporting Information). Additionally, the *A. excavata* host genome encodes for at least one GH18 chitinase, a GH20 chitobiase and an AA15 lytic chitin and cellulose monooxygenase (Table S3, Supporting Information). This host-symbiont system may thus rely on relevant biomass-converting enzymes that are different from those occurring in other systems known so far (Horn *et al*. 2012; Hemsworth *et al*. 2015). Experimental incubation in aquaria demonstrated that *A. excavata* assimilated ^13^C-labelled carbon from both diatom organic matter and bacteria (Maier *et al*. 2020).

**Figure 5.**
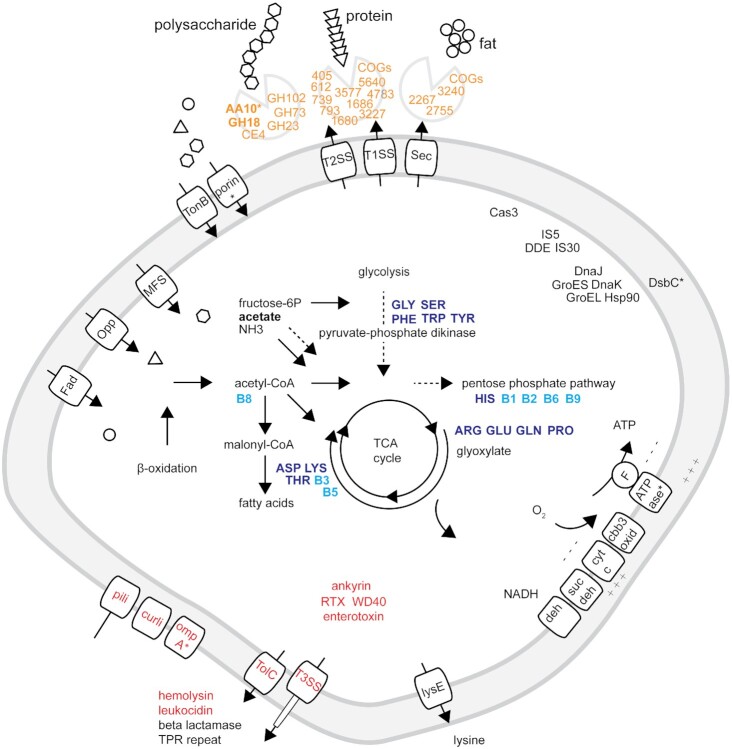
Selected functions of the ‘*Candidatus* Acestibacter aggregatus’ as inferred from genome comparisons. The depicted cell is hypothesized to secrete enzymes active in the degradation of recalcitrant compounds that fuel a nutritional symbiosis. Enzyme activities are predicted towards structural polysaccharides from zooplankton exoskeletons (chitin), algae cell walls (xylan and cellulose) and bacteria cell walls (murein) as well as towards organism's tissues (protein) and storage materials (fat). Depolymerization may also occur in the periplasmic space comparable to the selfish mode of polysaccharide utilization by free-living marine bacteria (Reintjes *et al*. 2019). Possible routes and modes of enzyme transport outside the depicted cell are unknown. The suggestions by OʼConnor *et al*. (2014) on the shipworm symbiont may apply. Cleavage products and enzymes become ingested with the food while some monomers are imported directly into the gill. The host may utilize amino acids and B-vitamins including whole symbiont cells and cleavage products like possibly acetate. Host access may be trough leakage, efflux proteins and lysosomal degradation. A dashed line indicates one or more genes only found in the host genome. An asterisk indicate proteins detected by proteomics. Bold highlight the minimal chitinolytic machinery, acetate and essential nutrients. Annotations are detailed in Table S1 (Supporting Information; symbiosis factors), Table S2 (Supporting Information; metabolism), Table 3 and Table S3 (Supporting Information; depolymerization) and Figure S4 (Supporting Information; amino acids and B-vitamins). The cell shape was outlined from a transmission electron microscopy image (Figure S1, Supporting Information). Visual inspection indicates a pleomorphic bacterium located in aggregates inside vacuoles scattered within the epithelial gill cells cytoplasm.

We hypothesize that ‘*Ca*. A. aggregatus’ utilizes structural polysaccharides (Fig. 5) such as bacterial peptidoglycan, algal cellulose and xylan, and copepod chitin as nutrient sources. If the enzymes are extracellular and secreted in the mucus coating the gill, they would be ingested along with the food particles. These enzymes could potentially contribute to extracellular digestion of biopolymers and thus assist the bivalve in exploiting various types of decaying organic matter.

## CONCLUSIONS

Our study provides insight into the functional potential of deep-water bivalve-associated Endozoicomonadaceae. Bacteria from this family have emerged as widespread associates of dense marine animal communities in vents, seeps, sediments and coral reefs, including fish and shellfish consumed by humans. The uncultured ‘*Ca*. A. aggregatus’ studied herein dominates the gill microbiome of *A. excavata* bivalves from deep-water coral reefs and a rock wall, mid-Norway. Genome analyses suggest a nutritional relationship, characterized by aerobic heterotrophy, synthesis of essential nutrients and the secretion of depolymerizing enzymes. The LPMO10 and GH18 minimal chitinolytic machinery identified is possibly involved in extracellular degradation processes that may assist the host in utilization of the more recalcitrant biopolymers available on the surrounding reef especially in winter. Eukaryote-like proteins, toxins and secretion systems likely support the intracellular symbiosis overall but the large genome does not exclude a free-living lifestyle and the relationship being facultative or less mutualistic. Under stressful conditions such as food shortage, heterotrophic symbionts could become pathogens. This ambiguity is supported by functions encoded in the Endozoicomonadaceae, which are similar to a putative mutualist in *Stylophora* coral (Bayer *et al*. 2013) and a parasite in *Bathymodiolus* mussels (Zielinski *et al*. 2009). However, the many repeats, transposons, and lowered mole % GC support that host and symbiont are becoming more integrated.

## Supplementary Material

fiab070_Supplemental_Files
