# Supplementary material for: Endozoicomonadaceae symbiont in gills of Acesta clam encodes genes for essential nutrients and polysaccharide degradation
Source: FEMS Microbiol Ecol. 2021 May 14;97(6):fiab070. doi: 10.1093/femsec/fiab070 (PMC8755941; doi:10.1093/femsec/fiab070)
Supplement: fiab070_Supplemental_Files [file fiab070_supplemental_files.zip › FigureS4_nutrients_revised_R2.docx]

A

*gdhA glnA arcC*

Oxoglutarate Glutamate (**Glu**) Glutamine (**Gln**)

*argF arcA*

Arginine (**Arg**)

*putA proC*

Glutamate Proline (**Pro**)

*aroFGH aroB aroQ aroE aroKL aroA aroC pheA aspB*

PEP Chorismate

*pheA phhA*

Phenylalanine (**Phe**) Tyrosine (**Tyr**)

*trpE trpD trpF trpC trpA*

Chorismate Tryptophan (**Trp**)

*aspB lysC*

Oxaloacetate Aspartate (**Asp**)

*asd dapA dapB dapD argD ~~dapE~~ dapF lysA*

Lysine (**Lys**)

*lysC asd hom thrB2 thrC tdh kbl*

Aspartate Threonine (**Thr**) Glycine

*prsA hisG hisE hisl hisA HisFH hisB hisC ~~hisN~~*

Ribose-5P

*hisD hisD*

Histidine (**His**)

*serA serC serB glyA*

Glycerate-3P Serine (**Ser**) Glycine (**Gly**)

B

*prsA purF purD purN purL purM thiC thiD*

Ribose-5P

*thiE*

Thiamine-P (**B1**)

*ribBA ribH ribE*

Ribulose-5P Riboflavin (**B2**)

*aspB nadB nadA nadC*

Oxaloacetate Nicotinate (**B3**)

*panD panC*

Aspartate Pantothenate (**B5**)

*epd pdxB serC pdxA pdxJ pdxH*

Erythrose-4P Pyridoxal-5P (**B6**)

*accA fabD bioC fabB fabG fabZ fabl fabB fabG fabZ*

Acetyl-CoA

*fabl bioH bioF bioA bioD bioB*

Biotin (**B8**)

*folE ~~phoAB~~ folB folK folP folC folA*

GTP glycolaldehyde Folate (**B9**)

Figure S4
