# Supplementary material for: Endozoicomonadaceae symbiont in gills of Acesta clam encodes genes for essential nutrients and polysaccharide degradation
Source: FEMS Microbiol Ecol. 2021 May 14;97(6):fiab070. doi: 10.1093/femsec/fiab070 (PMC8755941; doi:10.1093/femsec/fiab070)
Supplement: fiab070_Supplemental_Files [file fiab070_supplemental_files.zip › Manuscript_Acestibacter_revised_Supplementary_Text_revised4.docx]

**Supplementary Text**

**Supplementary MATERIALS AND METHODS**

**Screening clams for "*Candidatus* Acestibacter aggregatus"**

The PCR for the restriction digest was performed on DNA from *Acesta excavata* gills and from a plasmid Ae2p1c4 containing a previously (Jensen *et al.* 2010) cloned 16S rRNA gene sequence from "*Ca.* A. aggregatus". The PCR reactions (15 µl) combined ~10 ng template DNA, 0.5 µM bacterial 16S rRNA gene primers 27f/1492r (Lane *et al.* 1991), 0.2 mM each dNTP, 1.5 mM MgCl_2_ and 0.2 U Taq DNA polymerase (New England BioLabs, USA). Reactions were performed on a Mastercycler Gradient machine (Eppendorf, Hamburg, Germany) with 98°C for 30 sec followed by 30 cycles of 98°C for 10 sec, 54°C for 25 sec, 72°C for 30 sec and a final 72°C for 2 min. Amplicons were digested with the 6-bp cutter *Sal*I (New England BioLabs, USA) and the restriction fragments profiled in an agarose gel (Fig. S1). DNA from a few profiles was checked for identity using Sanger sequencing. These amplicons were cleaned by the NucleoSpin Gel and PCR Cleanup kit (Macherey-Nagel, Düren, Germany) and sequenced by GATC Biotech (Eurofins Genomics, Germany). The Sanger sequences were manually read from the chromatograms.

**Enrichment by cell fractionation**

Initial attempts to enrich for bacterial DNA using cell fractionation (for principle see e.g. Ponnudurai *et al.* 2017) were not pursued until a well-defined separation of "*Ca*. A. aggregatus" symbiont bacteria and *Acesta excavata* host cells had been achieved. For example, following centrifugation for 10 minutes (188 × *g* swing out rotor) of a 1 ml gill suspension (0.07 g ml^-1^ dounce homogenised in PBS pH 7.2) layered on a 10 ml sucrose solution (50%), the top orange fraction smeared towards the white bottom fraction. There was also concern of loss from unbroken symbiont aggregates and aggregates inside unbroken host cells sedimenting along with other host cells and debris. Instead, the NEBNext microbiome DNA enrichment kit (New England BioLabs, USA) was used. To indicate bacterial DNA enrichment efficiency (Fig. S1), the Ae24 DNA was serially diluted, and PCR amplified using bacterial primers 27f/1492r (Lane 1991) and eukaryal primers EukF/EukR (DeLong 1992). The PCR reactions were performed as for the screening. Following agarose gel electrophoresis band intensity was evaluated to indicate the relative content of bacterial DNA (Fig. S1).

**Binning**

Briefly, having located the dominant "*Ca*. A. aggregatus" phylotype (in contig 29) using a previously recovered 16S rRNA gene sequence (clone Ae2p1d1), a sum of recommended 100-150 kb (Patil, Roune and McHardy 2012) of contigs of similar 31x coverage and 37.6 mole % GC to contig 29 were selected, if found by hmmscan v 3.1b2 (http://hmmer.org) to include any of the amphora single copy marker genes (Wu and Eisen 2008). Seven contigs were found (~135 kb), all with a different marker (*rpmA*, *dnaG*, *rplN*, *rplL*, *rplT*, *rplS*, *pgk*) and all affiliated with the order Oceanospirillales. Potentially contaminating genomes were downloaded from GenBank. These represented (taxid) the verrucomicrobium *Rubritalea marina* (361055) and the actinobacterium *Smaragdicoccus* *niigatensis* (359359) both indicated present by the amplicon sequencing. As no *Acesta* clam was found genome sequenced, the molluscs were represented by the bivalves *Crassostrea gigas* (29159), *Mytilus galloprovincialis* (29158) and *Dreissena polymorpha* (45954), the snails *Lottia gigantea* (225164) and *Biomphalaria glabrata* (6526), and the slug *Aplysia californica* (6500). The Eukarya were further represented by the foraminifera *Astrammina rara* (46078) and *Reticulomyxa filosa* (46433), the annelida *Capitella teleta* (283909), and the echinodermata *Ophiothrix spiculata* (1266684).

**Supplementary RESULTS AND DISCUSSION**

**Host adapted functions**

A larger set of 430 COGs represented "*Ca*. A. aggregatus" gene abundances >2-fold enriched above the average Endozoicomonadaceae (normalised dataset). Some of these COGs are included among the functions selected for being potentially associated with host-symbiont interaction (Table S1). In "*Ca*. A. aggregatus", ankyrin repeats (COG0666) were 7-fold enriched relative to the average Endozoicomonadaceae. The ankyrins affiliated with *Crassostrea* *gigas* (55% identity) and *Endozoicomonas* *acroporae* (35% identity) suggesting these are eukaryote like proteins. Ankyrins were named after the human membrane-associated ankyrin protein that attach the cytoskeleton to the plasma membrane and were first thought an exclusively eukaryotic protein family (Al-Khodor *et al*. 2010 and references herein). With complete genome sequencing, however, ankyrins were found in all domains of life including viruses. Encoded in bacterial symbionts they may, for example, mimic or manipulate host functions to reinforce the association by mediating direct protein-protein interactions. They may subvert host defence and protect against leucocytes and other immune cells of the hosts. Heterologously expressed in *Escherichia coli* ankyrins inhibited phagocytosis in sponge amoeba cells (Nguyen, Liu and Thomas 2014).

The LPMO catalytic domain (COG3397) was 10-fold enriched relative to the average Endozoicomonadaceae. The “*Ca*. A. aggregatus” LPMO is annotated by IMG as a chitin-binding protein. Folders *et al*. (2000) studied a homologous protein from *Pseudomonas aeruginosa*, demonstrated its binding to chitin and proposed to name it Chitin binding protein D (CbpD). CbpD is one of the major proteins secreted by many clinical isolates of *P. aeruginosa* and most of these proteins contribute to virulence. CbpD was recently studied by Askarin *et al*. (2021) who found that CbpD from *P. aeruginosa* is a chitin-oxidizing virulence factor that promotes survival of the bacterium in human blood by attenuating the terminal complement cascade. The substrate for the catalytic domain of CbpD in the human body appears to remain unknown. However, this supports that LPMOs may have multiple roles and provides insight on the host-symbiont integration. CbpD is represented by *P.* *aeruginosa* in the phylogenetic tree (Fig. 4).

A 10-fold enrichment was also observed for the RTX repeats (COG2931). RTX are a large group of pore-forming toxins that includes hemolysins (Linhartová *et al.* 2010). Some of the toxins have signal peptides suggesting they are secreted. The secretion systems of a HlyD family T1SS (COG0845) indicated for secretion of RTX from the algicidal *Hahella* (Jeong *et al.* 2005) and from the *Bathymodiolus* gill symbionts (Sayavedra *et al.* 2015) were also indicated for the "*Ca*. A. aggregatus". T2SS and T3SS, which are both significantly enriched in Endozoicomonadaceae (Fig. S3), were also indicated for “*Ca*. A. aggregatus”. T3SS is a needle-like system used by pathogenic bacteria to inject toxins into host cells. Other repeats such as the tetratricopeptide repeat TPR (COG0790) was 13-fold enriched while the tryptophan aspartic acid WD40 repeat (COG2319) was <2-fold enriched.

The transposons and IS elements represented a sum of 54 genes in the "*Ca*. A. aggregatus" compared to for example six genes in the free living *Neptunimonas japonica* (Fig. 3B). The IS5 (COG3039) and IS30 (COG2826) transposons were 5 to 9-fold enriched in COG relative abundances (Fig. 3B). Transposons may have contributed to the extended branch length of "*Ca*. A. aggregatus" in the phylogenetic tree (Fig. 2). The COGs of chaperones such as DnaJ and GroES, were also relatively abundant (Table S1).

**Amino acids and B-vitamin synthesis**

The nutrients may be transferred to *A. excavata* by modes of 'farming', 'milking' and 'leakage' as suggested to describe carbon transfer from microorganisms to their bivalve hosts in autotrophic symbioses (Goreau, Goreau and Yonge 1973; Streams, Fisher and Fiala-Médioni 1997). Potentially, *A. excavata* feeds on the "*Ca.* A. aggregatus" production of essential nutrients and biomass while the "*Ca*. A. aggregatus" suppress interference, although leaking some compounds and being digested when senescent. Recently, more evidence for these modes was revealed from the *B. azoricus* mussel symbiosis. The *B. azoricus* host was indicated dependent on its sulfur- and methane oxidising gill symbionts for supply with amino acids and cofactors (Ponnundurai *et al.* 2017, 2020). In a way similar to the *B. azoricus*, 'farming' (symbiont degradation) may predominate nutrient transfer in *A. excavata* and involve a host lysosomal cystein protease cathepsin (COG4870) expressed by six peptides. Previous electron microscopy revealed curled up structures that might have been remains of "*Ca.* A. aggregatus" cell membranes but the visual evidence for symbiont degradation was uncertain. Perhaps less relevant is 'milking' (carbon translocation), which may, as indicated for the *Osedax* symbiont (Goffredi *et al.* 2014), involve a lysine efflux protein LysE (COG1280) detected by three genes in "*Ca*. A. aggregatus". Lysine export across the outer membrane appears poorly understood (Jones *et al.* 2015). To summarise, all these potential interactions are probably part of a tight host control on the aggregate as suggested from their regular size and distribution. Given that a starved or otherwise stressed heterotrophic symbiont may turn its enzymatic arsenal against its host, it may be speculated that host control involve restricting the symbiont to inhabit distinct tissue locations. The "*Ca*. A. aggregatus" symbiont is hosted as microcolonies (aggregates) inside the cytoplasm of some epithelial cells in the gill filaments.

**Heterotrophic central metabolism**

Genes were not found in the genome of “*Ca*. A. aggregatus”, for the Calvin cycle key enzyme ribulose-1,5-bisphosphate carboxylase nor for the reverse TCA cycle key enzymes fumarate reductase, alpha-oxoglutarate synthase and ATP-citrate lyase (Gottshalk 1988). Absence of CO_2_ assimilation was supported by a failure to find genes indicating energy from sulfur oxidation (*soxBYZ*) as well as the adenosine-5'-phosphosulfate reductase (*aprA*), particulate methane monooxygenase (*pmoA*) and methanol dehydrogenase (*mxaF*). Previous PCR attempts targeting the genes *aprA*, *pmoA* and *mxaF* were also negative (Jensen *et al.* 2010). The genome neither revealed hydrogenases (*hupL* genes) or genes for anaerobic respiration with nitrogen, such as *nar, nir, nor* and *nos* (Table S2). As the "*Ca*. A. aggregatus" genome is not closed, undetected genes cannot be excluded. Therefore, supplementary oxidation and assimilation may provide some nutritional benefit to hosts (Hovland, Jensen and Indreiten 2012; Vohsen *et al.* 2020; Goffredi *et al.* 2020). Additionally, some of our blastp checked genes showed relationship with genes from organisms in seep environments such as gene 10004371 that revealed 63% identity with a *lysE* family translocator of a Methylococcales symbiont of a *Iophon* sponge from the deep-sea asphalt volcano Chapopote Knoll in the Gulf of Mexico (KAF3978697; Table S1). Furthermore, there were genes of uncertain annotation and genes encoding proteins of uncertain activity that might contribute to sustenance such as the carbohydrate auxiliary activity families AA3 and AA4 (Table 3; Table S3).

The utilization of recalcitrant polysaccharides may require alternative pathways (anapleurotic) to feed substrates into the central carbohydrate metabolism. Along these lines it was for example indicated that the degradation of propionyl-CoA, from beta-oxidation of odd-numbered fatty acids, missed at least one gene. Furthermore, propionyl-CoA oxidation via the methylcitrate cycle was lacking the gene encoding methylisocitrate lyase to form pyruvate and succinate (Dolan *et al.* 2018) and more genes were missing for oxidation to either pyruvate or succinyl-CoA (Gottschalk 1988). The malic enzyme was found encoded (EC:1.1.1.40) hence pyruvate may be formed from malate. Ethanol may be formed from acetate (EC:1.2.1.3, EC:1.1.1.2). Anaplerotic pathways and the ability to use short intermediates could benefit the intracellular lifestyle (Goffredi *et al.* 2014). Whether intermediates such as chitin derived acetate may be utilized by the *A. excavata* host seems plausible but remains an interesting question.

**References**

Al-Khodor S, Price CT, Kalia A *et al*. Ankyrin-repeat containing proteins of microbes: a conserved structure with funcitonal diversity *Trends Microbiol* 2010; **18:** 132-139.

Askarian F, Uchiyama S, Masson H et al. The lytic polysaccharide monooxygenase CbpD promotes *Pseudomonas aeruginosa* virulence in systemic infection. Nat Commun 2021, **12:**1230 doi.org/10.1038/s41467-021-21473-0

DeLong EF. Archaea in coastal marine environments *P Natl Acad Sci USA* 1992; **89:** 5685-5689.

Dolan SK, Wijaya A, Geddis SM *et al.* Loving the poison: the methylcitrate cycle and bacterial pathogenesis *Microbiology* 2018; **164:** 251-259.

Folders J, Tommassen J, van Loon LC *et al*. Identification of a chitin-binding protein secreted by *Pseudomonas aeruginosa*. J Bacteriol 2000; **182:** 1257-1263.

Gottschalk G. *Bacterial Metabolism*. Second edition. New-York: Springer-Verlag, 1988.

Goffredi SK, Yi H, Zhang Q *et al*. Genomic versatility and functinal variation between two dormant heterotrophic symbionts of deep-sea *Osedax* worms *ISME J* 2014; **8:** 908-924.

Goffredi SK, Motooka C, Fike DA *et al.* Facultative chemosynthesis in a deep-sea anemone from hydrothermal vents in the Pescadero Basin, Gulf of California *bioRxiv* 2020 doi: https://doi.org/10.1101/2020.08.10.245456.

Goreau TF, Goreau NI, Yonge CM. On the utilization of photosynthetic products from zooxanthellae and of a dissolved amino acid in *Tridacna maxima* f *elongata* (Mollusca: Bivalvia)* *J Zool Lond* 1973; **169:** 417-454.

Hovland M, Jensen S, Indreiten T. Unit pockmarks associated with *Lophelia* coral reefs off mid-Norway: more evidence of control by ‘fertilizing’ bottom currents Geo Mar Lett 2012; 32: 545-554.

Jensen S, Duperron S, Birkeland NK *et al*. Intracellular Oceanospirillales bacteria inhabit gills of *Acesta bivalves* *FEMS Microbiol Ecol* 2010: **74:** 523-533.

Jones CM, Hernández Lozada NJ, Pfleger BF. Efflux systems in bacteria and their metabolic engineering applications *Appl Microbiol Biotechnol* 2015; **99:** 9381-9393.

Jeong H, Yim JH, Lee C *et al*. Genomic blueprint of *Hahella chejuensis*, a marine microbe producing an algicidal agent *Nucleic Acids Res* 2005; **33:** 7066-7073.

Lane DJ. 16S/23S rRNA sequencing. In: Stackebrandt E, Goodfellow M (ed.). *Nucleic Acid Techniques in Bacterial Systematics*. UK: Chichester, JohnWiley & Sons, 1991, 115-175.

Linhartová I, Bumba L, Maín J *et al*. RTX proteins: a highly diverse family secreted by a common mechanism *FEMS Microbiol Rev* 2010; **34:** 1076-1112.

Nguyen MTHD, Liu M, Thomas T. Ankyrin-repeat proteins from sponge symbionts modulate amoebal phagocytosis *Mol Ecol* 2014; **23:** 1635-1645.

Patil KR, Roune L, McHardy AC. The PhyloPythiaS web server for taxonomic assignment of metagenome sequences *PLoS One* 2012; **7:** e38581.

Ponnudurai R, Kleiner M, Sayavedra L *et al.* Metabolic and physiological interdependencies in the *Bathymodiolus azoricus* symbiosis *ISME J* 2017; **11:** 463-477.

Ponnudurai R, Heiden SE, Sayavedra L *et al*. Comparative proteomics of related symbiotic mussel species reveals high variability of host–symbiont interactions *ISME J* 2020; **14:** 649-656.

Sayavedra L, Kleiner M, Ponnudurai R *et al*. Abundant toxin-related genes in the genomes of beneficial symbionts from deep-sea hydrothermal vent mussels *Elife* 2015; 4.

Streams ME, Fisher CR, Fiala-Médioni A. Methanotrophic symbiont location and fate of carbon incorporated from methane in a hydrocarbon seep mussel *Mar Biol* 1997; **129:** 465-476.

Vohsen SA, Gruber-Vodicka HR, Osman EO *et al.* Deep-sea corals near cold seeps associate with chemoautotrophic bacteria that are related to the symbionts of cold seep and hydrothermal vent mussels *bioRxiv* 2020 doi: https://doi.org/10.1101/2020.02.27.968453.

Wu M, Eisen JA. A simple, fast, and accurate method of phylogenomic inference *Genome Biol* 2008; **9:** R151.
