# Supplementary material for: Endozoicomonadaceae symbiont in gills of Acesta clam encodes genes for essential nutrients and polysaccharide degradation
Source: FEMS Microbiol Ecol. 2021 May 14;97(6):fiab070. doi: 10.1093/femsec/fiab070 (PMC8755941; doi:10.1093/femsec/fiab070)
Supplement: fiab070_Supplemental_Files [file fiab070_supplemental_files.zip › Supplementary_Legends.docx]

**Table S1**. Selected functions potentially associated with symbiont-host interactions as found in the gene set of "*Candidaus* Acestibacter aggregatus". Gene count is shown in comparison with genomes from 27 gammaproteobacterial references.

**Table S2.** Central metabolic pathways deduced from the "*Candidatus* Acestibacter aggregatus" gene set. (A) Oxidative phosphorylation. (B) Glycolysis. (C) Tricarboxylic acid cycle. (D) Pentose phosphate cycle. (E) Sulfur metabolism. (F) Nitrogen metabolism. (G) Beta-oxidation. (H) Supplementary oxidation and assimilation. The pathways are based on functions named in the gene set functional profile 'KEGG Pathways via EC'.

**Table S3.** Depolymerising enzyme profiles deduced from the "*Candidatus* Acestibacter aggregatus" gene set and the full metagenome gene set. (A) Carbohydrate depolymerisation. (B) Protein depolymerisation. (C) Peptide depolymerisation. (D) Lipid depolymerisation. The functions are based on searches with the metagenome in dbCAN2 (Zhang *et al.* 2018) for carbohydrate active enzymes and in IMG ER (Markowitz *et al.* 2014) for 'protease', 'peptidase' and 'lipase' in the gene set functional COG profile. Complete host hits are shown only for the carbohydrate active enzymes.

**Figure S1.** Assessment of aggregates and DNA. (A) aggregates visualised from a drawing made while screening gill tissues by microscopy. They can be compared with confocal and electron microscopy images prepared during the previous study (Jensen *et al.* 2010). In red, gill tissue subjected to fluorescent *in situ* hybridisation with the bacterial probe Eub338 (Ae7). The same image is seen to the left (no filter) and to the right (dapi filter). Note the close proximity of aggregates to the gill filament surface. (B-D) DNA visualised by 1-2% agarose gel electrophoresis. (B) Extracted DNA from clam Ae24 prior to genome sequencing (1), prior to NEBNext enrichment (2) and prior to pooled (3). (C) PCR amplified Ae24 DNA using bacterial 16S rRNA gene primers 27f/1492r (upper photo) and using eukaryal 18S rRNA gene primers EukF/EukR (DeLong 1992) (lower photo). To indicate the amount of bacterial vs eukaryal DNA, the DNA (13 ng) was serially three fold diluted (lanes 1-7). The PCR was performed as for the restriction digest. Included are representative *Sal*I restriction profiles showing amplicons of Ae24 (d) and profile (d’) and control Ae2p1d1 (e, e’). (D) Sequenced microbiome amplicons as represented by Ae23 (1) and Ae24 (3), including amplicons of the Ae24 pooled (2). PCR controls were *E. coli* (a), host muscle tissue Ae18 (b) and water (c). M is 1 kb Fermentas marker (~400 ng). In (F), DNA quality is assessed using the Qubit fluorometer (concentration) and the Nanodrop spectrophotometer (ratios).

**Figure S2.** Rarefaction curves from simultaneously amplified V3-V4 regions of Bacteria and Archaea 16S rRNA gene sequences from microbiomes associated with gills of the *Acesta excavata* clams (prefixed Ae). The sequences were in mothur (Schloss *et al.* 2009) ascribed to operational taxonomic units (OTUs) at 97% sequence identity.

**Figure S3.** Differential gene abundance in COGs between Endozoicomonadaceae (n=11) and other Gammaproteobacteria (n=17). Bold indicate COGs represented by the "*Candidatus* Acestibacter aggregatus". An asterisk indicate "*Ca.* A. aggregatus" gene abundance >2-fold above the average gene abundance of the other Endozoicomonadaceae. LEfSe was used to determine the statistical significance and the effect size of the COGs. The genomes were normalised to 1107 genes each, resulting in a data set of in total 2631 COGs.

**Figure S4.** Biosynthetic pathways for amino acid and B-vitamin synthesis deduced from the "*Candidatus* Acestibacter aggregatus" gene set. The pathways are represented by arrows that each indicates one step catalysed by the enzyme of the named gene. There are three steps for which no gene was found (strikethrough) and there are several steps with more than one gene (additional genes not shown for clarity). Missing genes may have evolved beyond recognition or they may be compensated for by genes present in the microbiome or in the host.
