# Supplementary material for: Endozoicomonadaceae symbiont in gills of Acesta clam encodes genes for essential nutrients and polysaccharide degradation
Source: FEMS Microbiol Ecol. 2021 May 14;97(6):fiab070. doi: 10.1093/femsec/fiab070 (PMC8755941; doi:10.1093/femsec/fiab070)
Supplement: fiab070_Supplemental_Files [file fiab070_supplemental_files.zip › TableS1_symbiosis_factors_R3.docx]

**Table S1.**

|  |  |  |  | Aligned | No. of genes | | |
| --- | --- | --- | --- | --- | --- | --- | --- |
| Gene id | COG | Function | Closest relative (accession no.) | (id %) | A | E | O |
| 10000085 | 0666 | Ankyrin | *Strongylocentrotus pur.* (XP030849476)^U^ | 599 (29) | 45 | 117 | 7 |
| 10000754 | 2826 | Transposase IS30 | *Psychromonas* sp. (WP137297870)^Fr^ | 103 (44) | 8 | 24 | 35 |
| 100005111 | 5048 | FOG: Zn-finger | *Crassostrea virginica* (XP022321223)^M^ | 291 (30) | 7 | 95 | 0 |
| 10000377 | 0845 | T1SS HlyD | *Kistimonas* gill symbiont (OQX37154)^M^ | 432 (55) | 5 | 102 | 141 |
| 10002062 | 2165 | T2SS PulG | *Kistimonas* gill symbiont (OQX38136)^M^ | 144 (73) | 5 | 33 | 26 |
| 10000981^1^ | 0790 | TPR Sel1 | *Pasteurella* sp. WM03 (WP135094552)^X^ | 192 (66) | 5 | 14 | 24 |
| 10001151 | 3039 | Transposase IS5 | *Arcobacter cryaeroph.* (WP081560999)^X^ | 182 (64) | 4 | 9 | 4 |
| 10004371 | 1280 | Efflux LysE | Methylococcales symb. (KAF3978697)^S^ | 130(63) | 3 | 44 | 100 |
| 10002248 | 2885 | OmpA | *Kistimonas* gill symbiont (OQX38305)^M^ | 169 (57) | 3 | 45 | 69 |
| 100002213 | 0484 | DnaJ | *Kistimonas* gill symbiont (OQX39668)^M^ | 379 (69) | 3 | 13 | 24 |
| 10001024^1^ | 3240 | Hemolycin | *Kistimonas* gill symbiont (OQX34880)^M^ | 303 (42) | 3 | 12 | 10 |
| 100000415 | 2931 | RTX-related | *Aeromonas rivipolle.* (WP106886437)^X^ | 843 (42) | 3 | 13 | 13 |
| 10001996 | 3637 | Membrane Porin | *Oleispira antarctica* (WP046008914)^Fr^ | 187 (40) | 2 | 20 | 10 |
| 10001183 | 1651 | DsbA, DsbC | *Zooshikella ganghwe.* (WP094788118)^Fr^ | 223 (44) | 2 | 26 | 22 |
| 100015613 | 1220 | Protease HslVU | *Pseudomonas* sp. (PTU02542)^Fr^ | 124 (72) | 2 | 10 | 16 |
| 10000638 | 0443 | DnaK | *Kistimonas* gill symbiont (OQX38881)^M^ | 619 (53) | 2 | 21 | 41 |
| 10002121 | 0459 | GroEL | *Pseudomonas monteilii* (TXI05247)^Fr^ | 057 (58) | 2 | 10 | 18 |
| 10002552 | 0234 | GroES | *Omnitrophica* bacter. (OGX38269)^Fr^ | 094 (39) | 2 | 11 | 18 |
| 100000514 | 00816^2^ | DNA binding H-NS | *Kistimonas* gill symbiont (OQX36710)^M^ | 139 (65) | 2 | 24 | 16 |
| 10003042^1^ | 12563^2^ | Hemolysin/Leukocid. | *Vibrio qinghaiensis* (WP094501882)^Fr^ | 601 (40) | 2 | 1 | 1 |
| 100000710 | 01609^2^ | Transposase DDE | Gammaproteobacterium (RLA58605)^Fr^ | 108 (58) | 2 | 147 | 89 |
| 10001964 | 4969 | Major pilin PilA | *Pseudomonas ang*. (WP090381737)^X^ | 143 (64) | 2 | 20 | 14 |
| 10000776 | 2319 | WD40 | *Rhizobium leg*. (WP130692856)^P^ | 454 (24) | 2 | 40 | 0 |
| 10000311 | 2718 | YeaH/YhbH sporulation | *Kistimonas* gill symbiont (OQX36615)^M^ | 424 (79) | 2 | 21 | 11 |
| 100001015 | 0312 | Zn protease TldD | *Kistimonas* gill symbiont (OQX38345)^M^ | 479 (76) | 2 | 26 | 20 |
| 10000351 | 0690 | Secretion SecE | *Haliea* sp. (MAL96078)^Fr^ | 119 (55) | 1 | 9 | 16 |
| 10001065^1^ | 1680 | Beta lactamase C | Candidate divis. KSB1 (KAA0229487)^Fr^ | 502 (50) | 1 | 13 | 30 |
| 10000564 | 2847 | Cu(I)-binding protein | *Kistimonas* gill symbiont (OQX38651)^M^ | 276 (35) | 1 | 10 | 15 |
| 10021556 | 7012^3^ | CRISPR Cas3 | *Methylovulum psychr*. (POZ51807)^Fr^ | 069 (64) | 1 | 3 | 8 |
| 10003346^1^ | 1462 | Curli protein CsgG | *Desulfobacter hydrog.* (WP111952520)^X^ | 231 (79) | 1 | 3 | 7 |
| 10457021 | 07906^2^ | Enterotoxin ShET2 | *Endozoicomonas acro.* (WP101747480)^C^ | 518 (26) | 1 | 6 | 0 |
| 10026483 | 0625 | Glutathione S-transf. | *Kistimonas* gill symbiont (OQX36904)^M^ | 209 (71) | 1 | 61 | 135 |
| 10001556 | 12161^2^ | HsdM | *Moritella viscosa* (WP075533112)^F^ | 386 (72) | 1 | 43 | 40 |
| 10068613 | 5405 | Protease HslVU | *Kistimonas* gill symbiont (OQX38677)^M^ | 179 (85) | 1 | 10 | 16 |
| 10000412 | 4122 | Methyltransfer. YrrM | *Kistimonas* gill symbiont (OQX35689)^M^ | 728 (52) | 1 | 13 | 8 |
| 100000412^1^ | 01037^2^ | Lrp/AsnC | Bdellovibrionales bacter. (OFZ16980)^X^ | 083 (65) | 1 | 37 | 90 |
| 100002216^1^ | 0860 | N-acetylmuramoyl-L-alanine amidase | *Kistimonas* gill symbiont (OQX39292)^M^ | 453 (62) | 1 | 11 | 17 |
| 10006491 | 4791 | T3SS EscT | *Kistimonas* gill symbiont (OQX38930)^M^ | 178 (76) | 1 | 10 | 3 |
| 10014782 | 0326 | HtpG (Hsp90) | *Kistimonas* gill symbiont (OQX35107)^M^ | 644 (68) | 1 | 10 | 19 |

A "*Ca*. A. aggregatus", E Other Endozoicomonadaceae (n = 10), O Other Gammaproteobacteria (n = 17),  ^M^mollusc, ^S^sponge, ^C^coral, ^F^fish, ^U^sea urchin, ^P^plant, ^Fr^free living, ^X^unclear, ^1^signal peptide, ^2^pfam, ^3^KO
