# Supplementary material for: Endozoicomonadaceae symbiont in gills of Acesta clam encodes genes for essential nutrients and polysaccharide degradation
Source: FEMS Microbiol Ecol. 2021 May 14;97(6):fiab070. doi: 10.1093/femsec/fiab070 (PMC8755941; doi:10.1093/femsec/fiab070)
Supplement: fiab070_Supplemental_Files [file fiab070_supplemental_files.zip › TableS2_metabolic_pathways_revised_R3.docx]

**Table S2. A. Oxidative phosphorylation.**

| Gene id | AA | Gene Product Name | Enzyme | COG |
| --- | --- | --- | --- | --- |
| 10006105 | 295 | 4-hydroxybenzoate polyprenyltransferase and related prenyltransferases | EC:2.5.1.- | COG0382 |
| 100000312 | 631 | Archaeal/vacuolar-type H+-ATPase subunit A | EC:3.6.3.14 | COG1155 |
| 100004614 | 166 | Cbb3-type cytochrome oxidase, subunit 1 | EC:1.9.3.1 | COG3278 |
| 10007485 | 160 | Cbb3-type cytochrome oxidase, subunit 1 | EC:1.9.3.1 | COG3278 |
| 10006401 | 515 | F0F1-type ATP synthase, alpha subunit | EC:3.6.3.14 | COG0056 |
| 10059962 | 460 | F0F1-type ATP synthase, beta subunit | EC:3.6.3.14 | COG0055 |
| 10006497 | 379 | Flagellar biosynthesis/type III secretory pathway ATPase | EC:3.6.3.14 | COG1157 |
| 100006211 | 103 | Heme/copper-type cytochrome/quinol oxidase, subunit 3 | EC:1.9.3.1 | COG1845 |
| 10002444 | 296 | Heme/copper-type cytochrome/quinol oxidase, subunit 3 | EC:1.9.3.1 | COG1845 |
| 10008775 | 166 | Heme/copper-type cytochrome/quinol oxidases, subunit 1 | EC:1.9.3.1 | COG0843 |
| 10008774 | 209 | Heme/copper-type cytochrome/quinol oxidases, subunit 1 | EC:1.9.3.1 | COG0843 |
| 10002446 | 523 | Heme/copper-type cytochrome/quinol oxidases, subunit 1 | EC:1.9.3.1 | COG0843 |
| 10002447 | 322 | Heme/copper-type cytochrome/quinol oxidases, subunit 2 | EC:1.9.3.1 | COG2010 |
| 10000626 | 51 | Heme/copper-type cytochrome/quinol oxidases, subunit 2 | EC:1.9.3.1 | COG1622 |
| 10002687 | 99 | hypothetical protein (ATP synthase in type III secretion protein N) | EC:3.6.3.14 | COG1157 |
| 100087710 | 50 | hypothetical protein (NADH-quinone oxidoreductase subunit L) | EC:1.6.5.3 | nd |
| 100006216 | 122 | hypothetical protein (NADH-quinone oxidoreductase subunit N) | EC:1.6.5.3 | COG1007 |
| 10001516 | 175 | Inorganic pyrophosphatase | EC:3.6.1.1 | COG0221 |
| 10008777 | 58 | NADH:ubiquinone oxidoreductase subunit 1 (chain H) | EC:1.6.5.3 | COG1005 |
| 10008776 | 108 | NADH:ubiquinone oxidoreductase subunit 1 (chain H) | EC:1.6.5.3 | COG1005 |
| 100006217 | 94 | NADH:ubiquinone oxidoreductase subunit 2 (chain N) | EC:1.6.5.3 | COG1007 |
| 100006219 | 115 | NADH:ubiquinone oxidoreductase subunit 4 (chain M) | EC:1.6.5.3 | COG1008 |
| 10000621 | 58 | NADH:ubiquinone oxidoreductase subunit 5 (chain L)/Multisubunit Na+/H+ antiporter, MnhA subunit | EC:1.6.5.3 | COG1009 |
| 10000622 | 165 | NADH:ubiquinone oxidoreductase subunit 5 (chain L)/Multisubunit Na+/H+ antiporter, MnhA subunit | EC:1.6.5.3 | COG1009 |
| 10000525 | 146 | Phosphohistidine phosphatase SixA | EC:3.1.3.- | COG2062 |
| 10000052 | 708 | Polyphosphate kinase | EC:2.7.4.1 | COG0855 |
| 10001736 | 301 | Polyprenyltransferase (cytochrome oxidase assembly factor) | EC:2.5.1.- | COG0109 |
| 10003877 | 198 | Rieske Fe-S protein | EC:1.10.2.2 | COG0723 |
| 10000572 | 237 | Succinate dehydrogenase/fumarate reductase, Fe-S protein subunit | EC:1.3.5.1 | COG0479 |
| 10000471 | 279 | Succinate dehydrogenase/fumarate reductase, flavoprotein subunit | EC:1.3.5.1 | COG1053 |
| 10000571 | 307 | Succinate dehydrogenase/fumarate reductase, flavoprotein subunit | EC:1.3.5.1 | COG1053 |

**Table S2. B. Glycolysis.**

| Gene id | AA | Gene product name | Enzyme | COG |
| --- | --- | --- | --- | --- |
| 100007812 | 386 | 3-phosphoglycerate kinase | EC:2.7.2.3 | COG0126 |
| 10000565 | 422 | 6-phosphofructokinase | EC:2.7.1.11 | COG0205 |
| 11171661 | 201 | Aldo/keto reductases, related to diketogulonate reductase | EC:1.1.1.2 | COG0656 |
| 10004083 | 376 | Enolase | EC:4.2.1.11 | COG0148 |
| 100007810 | 352 | Fructose/tagatose bisphosphate aldolase | EC:4.1.2.13 | COG0191 |
| 10017613 | 231 | Glyceraldehyde-3-phosphate dehydrogenase/erythrose-4-phosphate dehydrogenase | EC:1.2.1.12 | COG0057 |
| 10788382 | 271 | Glyceraldehyde-3-phosphate dehydrogenase/erythrose-4-phosphate dehydrogenase | EC:1.2.1.12 | COG0057 |
| 10000333 | 475 | Male sterility protein | EC:1.2.1.- | COG3320 |
| 10001827 | 507 | NAD-dependent aldehyde dehydrogenases | EC:1.2.1.- | COG1012 |
| 100001511 | 455 | NAD-dependent aldehyde dehydrogenases | EC:1.2.1.8 | COG1012 |
| 10000123 | 518 | Phosphoglyceromutase | EC:5.4.2.12 | COG0696 |
| 10623731 | 329 | Phosphomannomutase | EC:5.4.2.2 | COG1109 |
| 10027063 | 87 | Phosphomannomutase | EC:5.4.2.2 | COG1109 |
| 10000107 | 164 | Phosphotransferase system mannitol/fructose-specific IIA domain (Ntr-type) | EC:2.7.1.69 | COG1762 |
| 100001713 | 853 | Pyruvate dehydrogenase complex, dehydrogenase (E1) component | EC:1.2.4.1 | COG2609 |
| 10002474 | 359 | Pyruvate/2-oxoglutarate dehydrogenase complex, dehydrogenase (E1) component, eukaryotic type, alpha subunit | EC:1.2.4.1 | COG1071 |
| 10002473 | 327 | Pyruvate/2-oxoglutarate dehydrogenase complex, dehydrogenase (E1) component, eukaryotic type, beta subunit | EC:1.2.4.1 | COG0022 |
| 10113942 | 76 | Pyruvate/2-oxoglutarate dehydrogenase complex, dihydrolipoamide acyltransferase (E2) component, and related enzymes | EC:2.3.1.12 | COG0508 |
| 10022134 | 436 | Pyruvate/2-oxoglutarate dehydrogenase complex, dihydrolipoamide acyltransferase (E2) component, and related enzymes | EC:2.3.1.12 | COG0508 |
| 10002471 | 294 | Pyruvate/2-oxoglutarate dehydrogenase complex, dihydrolipoamide acyltransferase (E2) component, and related enzymes | EC:2.3.1.12 | COG0508 |
| 10000575 | 480 | Pyruvate/2-oxoglutarate dehydrogenase complex, dihydrolipoamide dehydrogenase (E3) component, and related enzymes | EC:1.8.1.4 | COG1249 |
| 10003226 | 280 | Triosephosphate isomerase | EC:5.3.1.1 | COG0149 |

**Table S2. C. Tricarboxylic acid cycle.**

| Gene id | AA | Gene product name | Enzyme | COG |
| --- | --- | --- | --- | --- |
| 10000573 | 944 | 2-oxoglutarate dehydrogenase complex, dehydrogenase (E1) component, and related enzymes | EC:1.2.4.2 | COG0567 |
| 10000965 | 864 | Aconitase B | EC:4.2.1.3 | COG1049 |
| 10000764 | 431 | Citrate synthase | EC:2.3.3.1 | COG0372 |
| 10000522 | 469 | Fumarase | EC:4.2.1.2 | COG0114 |
| 10788381 | 82 | hypothetical protein (malate dehydrogenase) | EC:1.1.1.37 | nd |
| 10002112 | 419 | Isocitrate dehydrogenases | EC:1.1.1.42 | COG0538 |
| 10132601 | 282 | Malate/lactate dehydrogenases | EC:1.1.1.37 | COG0039 |
| 10002167 | 468 | Predicted dehydrogenase - COG0579 | EC:1.1.5.4 | COG0579 |
| 100001713 | 853 | Pyruvate dehydrogenase complex, dehydrogenase (E1) component | EC:1.2.4.1 | COG2609 |
| 10002474 | 359 | Pyruvate/2-oxoglutarate dehydrogenase complex, dehydrogenase (E1) component, eukaryotic type, alpha subunit | EC:1.2.4.1 | COG1071 |
| 10002473 | 327 | Pyruvate/2-oxoglutarate dehydrogenase complex, dehydrogenase (E1) component, eukaryotic type, beta subunit | EC:1.2.4.1 | COG0022 |
| 10113942 | 76 | Pyruvate/2-oxoglutarate dehydrogenase complex, dihydrolipoamide acyltransferase (E2) component, and related enzymes | EC:2.3.1.12 | COG0508 |
| 10022134 | 436 | Pyruvate/2-oxoglutarate dehydrogenase complex, dihydrolipoamide acyltransferase (E2) component, and related enzymes | EC:2.3.1.12 | COG0508 |
| 10000574 | 406 | Pyruvate/2-oxoglutarate dehydrogenase complex, dihydrolipoamide acyltransferase (E2) component, and related enzymes | EC:2.3.1.61 | COG0508 |
| 10002471 | 294 | Pyruvate/2-oxoglutarate dehydrogenase complex, dihydrolipoamide acyltransferase (E2) component, and related enzymes | EC:2.3.1.12 | COG0508 |
| 10000575 | 480 | Pyruvate/2-oxoglutarate dehydrogenase complex, dihydrolipoamide dehydrogenase (E3) component, and related enzymes | EC:1.8.1.4 | COG1249 |
| 10000572 | 237 | Succinate dehydrogenase/fumarate reductase, Fe-S protein subunit | EC:1.3.5.1 | COG0479 |
| 10000471 | 279 | Succinate dehydrogenase/fumarate reductase, flavoprotein subunit | EC:1.3.5.1 | COG1053 |
| 10000571 | 307 | Succinate dehydrogenase/fumarate reductase, flavoprotein subunit | EC:1.3.5.1 | COG1053 |
| 10000577 | 288 | Succinyl-CoA synthetase, alpha subunit | EC:6.2.1.5 | COG0074 |
| 10000576 | 390 | Succinyl-CoA synthetase, beta subunit | EC:6.2.1.5 | COG0045 |

**Table S2. D. Pentose phosphate cycle.**

| Gene id | AA | Gene product name | Enzyme | COG |
| --- | --- | --- | --- | --- |
| 10000565 | 422 | 6-phosphofructokinase | EC:2.7.1.11 | COG0205 |
| 100007810 | 352 | Fructose/tagatose bisphosphate aldolase | EC:4.1.2.13 | COG0191 |
| 10466041 | 52 | hypothetical protein (deoxyribose-phosphate aldolase) | EC:4.1.2.4 | nd |
| 10010041 | 223 | Pentose-5-phosphate-3-epimerase | EC:5.1.3.1 | COG0036 |
| 10623731 | 329 | Phosphomannomutase | EC:5.4.2.2 | COG1109 |
| 10027063 | 87 | Phosphomannomutase | EC:5.4.2.2 | COG1109 |
| 10003055 | 311 | Phosphoribosylpyrophosphate synthetase | EC:2.7.6.1 | COG0462 |
| 10000107 | 164 | Phosphotransferase system mannitol/fructose-specific IIA domain (Ntr-type) | EC:2.7.1.69 | COG1762 |
| 10162601 | 121 | Ribose 5-phosphate isomerase | EC:5.3.1.6 | COG0120 |
| 10021114 | 95 | Ribose 5-phosphate isomerase | EC:5.3.1.6 | COG0120 |
| 10000264 | 668 | Transketolase | EC:2.2.1.1 | COG0021 |

**Table S2. E. Sulfur metabolism.**

| Gene id | AA | Gene product name | Enzyme | COG |
| --- | --- | --- | --- | --- |
| 10001296 | 274 | 3'(2'), 5'-Bisphosphate nucleotidase | EC:3.1.3.7 | COG1218 |
| 10006105 | 295 | 4-Hydroxybenzoate polyprenyltransferase | EC:2.5.1.39 | COG0382 |
| 100013012 | 242 | Lipopolysaccharide export system ATP-binding protein | EC:3.6.3.- | COG1137 |
| 10001286 | 239 | Lipoprotein-releasing system ATP-binding protein | EC:3.6.3.- | COG1136 |
| 10001733 | 273 | Zinc transport system ATP-binding protein | EC:3.6.3.- | COG1121 |
| 10000025 | 602 | ATP-binding cassette, subfamily B, bacterial MsbA | EC:3.6.3.- | COG1132 |
| 10880542 | 96 | Alkanesulfonate monooxygenase | EC:1.14.14.5 | nd |
| 10001975 | 320 | MoxR-like ATPase | EC:3.6.3.- | COG0714 |
| 10001736 | 301 | Protoheme IX farnesyltransferase | EC:2.5.1.- | COG0109 |
| 10001694 | 275 | Thiosulfate/3-mercaptopyruvate sulfurtransferase | EC:2.8.1.1 2.8.1.2 | COG2897 |

**Table S2. F. Nitrogen metabolism.**

| Gene id | AA | Gene product name | Enzyme | COG |
| --- | --- | --- | --- | --- |
| 100013012 | 242 | ABC-type (unclassified) transport system, ATPase component | EC:3.6.3.- | COG1137 |
| 10001286 | 239 | ABC-type antimicrobial peptide transport system, ATPase component | EC:3.6.3.- | COG1136 |
| 10001733 | 273 | ABC-type Mn/Zn transport systems, ATPase component | EC:3.6.3.- | COG1121 |
| 10000025 | 602 | ABC-type multidrug transport system, ATPase and permease components | EC:3.6.3.- | COG1132 |
| 100000319 | 313 | Carbamate kinase | EC:2.7.2.2 | COG0549 |
| 10461681 | 216 | Carbonic anhydrase | EC:4.2.1.1 | COG0288 |
| 100000323 | 459 | Glutamate dehydrogenase/leucine dehydrogenase | EC:1.4.1.4 | COG0334 |
| 10000678 | 471 | Glutamine synthetase | EC:6.3.1.2 | COG0174 |
| 10001975 | 320 | MoxR-like ATPases | EC:3.6.3.- | COG0714 |
| 10002391 | 1148 | NAD-specific glutamate dehydrogenase | EC:1.4.1.2 | COG2902 |
| 10004222 | 1618 | NAD-specific glutamate dehydrogenase | EC:1.4.1.2 | COG2902 |
| 10003971 | 477 | NAD-specific glutamate dehydrogenase | EC:1.4.1.2 | COG2902 |
| 100001016 | 277 | Predicted amidohydrolase - COG0388 | EC:3.5.5.1 | COG0388 |

**Table S2. G. Beta-oxidation.**

| Gene id | AA | Gene Product Name | Enzyme | COG |
| --- | --- | --- | --- | --- |
| 10000431 | 444 | Long-chain fatty acid transport protein (*fadL*) |  | COG2067 |
| 10000765 | 682 | NADH:flavin oxidoreductases (*fadH*) | EC:1.3.1.34 | COG1902 |
| 10001113 | 400 | Acetyl-CoA acetyltransferase (*atoB*) | EC:2.3.1.9 | COG0183 |
| 10000853 | 393 | Acetyl-CoA acetyltransferase (*fadA*) | EC:2.3.1.16 | COG0183 |
| 10000854 | 722 | 3-hydroxyacyl-CoA dehydrogenase (*fadB*) | EC:1.1.1.35 | COG1250 |
| 10000287 | 662 | Acyl-coenzyme A synthetases/AMP-(fatty) acid ligases (*fadD*) | EC:6.2.1.16 | COG0365 |
| 10000074 | 598 | Acyl-CoA dehydrogenases (*fadE*) |  | COG1960 |

**Table S2. H. Supplementary oxidation and assimilation (less clear).**

| Gene id | AA | Gene Product Name | Enzyme | COG |
| --- | --- | --- | --- | --- |
| 10002463 | 96^1^ | Cytochrome P450 |  | pfam00067 |
| 11025552 | 183^1^ | Cytochrome P450 |  | pfam00067 |
| 10002462 | 65^1^ | Cytochrome P450 |  | pfam00067 |
| 10411982 | 48^1^ | Cytochrome P450 |  | pfam00067 |
| 10004437 | 325 | Biotin-(acetyl-CoA carboxylase) ligase | EC:6.3.4.15 | COG1654 |
| 10880542 | 96^1^ | Hypothetical protein | EC:1.14.14.5 | K04091 |
| 10001733 | 273 | ABC transporter | EC:3.6.3.- | COG1121 |

^1^pfam or KO
