# Supplementary material for: Endozoicomonadaceae symbiont in gills of Acesta clam encodes genes for essential nutrients and polysaccharide degradation
Source: FEMS Microbiol Ecol. 2021 May 14;97(6):fiab070. doi: 10.1093/femsec/fiab070 (PMC8755941; doi:10.1093/femsec/fiab070)
Supplement: fiab070_Supplemental_Files [file fiab070_supplemental_files.zip › TableS3_depolymerisation.docx]

**Table S3. A. Carbohydrate depolymerisation.**

| Gene id | CAZy | COG | Gene product name | Symbiont | Host |
| --- | --- | --- | --- | --- | --- |
| 10000305^1^ | GH23 | COG1388 | LysM repeat (additionally COG0714, COG4623) | 3 | 0 |
| 10000717 | GT9 | COG0859 | ADP-heptose:LPS heptosyltransferase | 2 | 0 |
| 10002353 | GT51 | COG5009 | Membrane carboxypeptidase/penicillin-binding protein | 2 | 0 |
| 100004811 | CE16 | COG3240 | Phospholipase/lecithinase/hemolysin (both COG3240) | 2 | 0 |
| 10000993 | GT30 | COG1519 | 3-deoxy-D-manno-octulosonic-acid transferase | 1 | 0 |
| 10000872^1^ | GH18 | COG3325 | Chitinase, GH18 family | 1 | 1 |
| 100003712 | AA3 | COG2303 | Choline dehydrogenase or related flavoprotein | 1 | 0 |
| 10000374 | AA4 | COG0277 | FAD/FMN-containing dehydrogenase | 1 | 0 |
| 10001862 | GT4 | COG0438 | Glycosyltransferase involved in cell wall bisynthesis | 1 | 1 |
| 10000162 | GT19 | COG0763 | Lipid A disaccharide synthetase | 1 | 0 |
| 10000317^1^ | GH102 | COG2821 | Membrane-bound lytic murein transglycosylase | 1 | 0 |
| 10004641 | GH103 | COG2951 | Membrane-bound lytic murein transglycosylase B | 1 | 0 |
| 10001412^1^ | CE4 | COG0726 | Peptidoglycan/xylan/chitin deacetylase, PgdA/CDA1 family | 1 | 0 |
| 10001957 | GH3 | COG1472 | Periplasmic beta-glucosidase and related glycosidases | 1 | 0 |
| 100001117 | GT2_2 | COG4261 | Predicted acyltransferase, LPLAT superfamily | 1 | 0 |
| 100000713^1^ | AA10 | COG3397 | Predicted carbohydrate-binding protein, contains CBM5 and CBM33 domains | 1 | 0 |
| 10000605 | CE11 | COG0774 | UDP-3-O-acyl-N-acetylglucosamine deacetylase | 1 | 0 |
| 100004210^1^ | GT28 | COG0707 | UDP-N-acetylglucosamine:LPS N-acetylglucosamine transferase | 1 | 0 |
| 10000014^1^ | GH73 | COG2992 | Uncharacterized FlgJ-related protein | 1 | 0 |
| 10715311 | GT11 | pfam01531 | Glyco_transf_11 - Glycosyl transferase family 11 | 1 | 5 |
| 10834291 | GT54 | pfam04666 | Glyco_transf_54 - N-Acetylglucosaminyltransferase-IV (GnT-IV) conserved region | 1 | 5 |
| 10507622 | GT31 | pfam01762 | Galactosyl_T - Galactosyltransferase | 0 | 33 |
| 10601251 | GT10 | pfam00852 | Glyco_transf_10 - Glycosyltransferase family 10 (fucosyltransferase) C-term | 0 | 18 |
| 10715461 | CE10 | COG2272 | Carboxylesterase type B | 0 | 9 |
| 10638082 | CBM47 | KO:K12373 | Fucose binding | 0 | 8 |
| 11132141 | GH31 | COG1501 | Alpha-glucosidase, glycosyl hydrolase family GH31 | 0 | 5 |
| 10828702 | AA15 | No data | Lytic cellulose and chitin monooxygenase | 0 | 3 |
| 10014571 | GH38 | COG0383 | Alpha-mannosidase | 0 | 2 |
| 11144961 | GT35 | COG0058 | Glucan phosphorylase | 0 | 2 |
| 10158862 | AA1_1 | COG2132 | Multicopper oxidase with three cupredoxin domains (includes cell division protein FtsP and spore coat protein CotA) | 0 | 2 |
| 10562491 | GT14 | pfam02485 | Branch - Core-2/I-Branching enzyme | 0 | 2 |
| 10569502 | GT47 | pfam03016 | Exostosin - Exostosin family | 0 | 2 |
| 10083051 | GT92 | pfam01697 | Glyco_transf_92 - Glycosyltransferase family 92 | 0 | 2 |
| 10332081 | GT27 | COG1215 | Glycosyltransferase, catalytic subunit of cellulose synthase and poly-beta-1,6-N-acetylglucosamine synthase | 0 | 1 |
| 10155322 | GT32 | COG3774 | Mannosyltransferase OCH1 or related enzyme | 0 | 1 |
| 10675611 | GT76 | COG5542 | Mannosyltransferase related to Gpi18 | 0 | 1 |
| 10698402 | GH20 | COG3525 | N-acetyl-beta-hexosaminidase | 0 | 1 |
| 10576762 | CBM14 | pfam01607 | CBM_14 - Chitin binding Peritrophin-A domain | 0 | 1 |
| 10579641 | CBM21 | pfam03370 | CBM_21 - Carbohydrate/starch-binding module (family 21) | 0 | 1 |
| 10683811 | CE13 | pfam03283 | PAE - Pectinacetylesterase | 0 | 1 |
| 10249091 | GT22 | pfam03901 | Glyco_transf_22 - Alg9-like mannosyltransferase family | 0 | 1 |
| 10603801 | GT49 | pfam13896 | Glyco_transf_49 - Glycosyl-transferase for dystroglycan | 0 | 1 |
| 10307763 | PL14_3 | No data | Alginate lyase | 0 | 1 |

^1^signal peptide (not predicted for host proteins)

**Table S3. B. Protein depolymerisation.**

| Gene id | COG | Gene product name | Symbiont | Host |
| --- | --- | --- | --- | --- |
| 10000437 | COG0330 | Regulator of protease activity HflC, stomatin/prohibitin superfamily | 3 | 2 |
| 10006841 | COG0466 | ATP-dependent Lon protease, bacterial type | 3 | 6 |
| 10005982^1^ | COG4783 | Putative Zn-dependent protease, contains TPR repeats | 3 | 4 |
| 10000947 | COG0312 | Predicted Zn-dependent protease or its inactivated homolog | 2 | 0 |
| 10000251 | COG0542 | ATP-dependent Clp protease ATP-binding subunit ClpA | 2 | 4 |
| 10114521 | COG0616 | Periplasmic serine protease, ClpP class | 2 | 0 |
| 10000676^1^ | COG0793 | C-terminal processing protease CtpA/Prc, contains a PDZ domain | 2 | 19 |
| 10068614 | COG1220 | ATP-dependent protease HslVU (ClpYQ), ATPase subunit | 2 | 0 |
| 10326112^1^ | COG3227 | Zn-dependent metalloprotease | 2 | 3 |
| 10222082^1^ | COG3577 | Predicted aspartyl protease | 2 | 17 |
| 10000738^1^ | COG5640 | Secreted trypsin-like serine protease | 2 | 8 |
| 10000707 | COG0465 | ATP-dependent Zn proteases | 1 | 9 |
| 10001716 | COG0501 | Zn-dependent protease with chaperone function | 1 | 2 |
| 10002314 | COG0705 | Membrane associated serine protease, rhomboid family | 1 | 0 |
| 10006844 | COG0740 | ATP-dependent protease ClpP, protease subunit | 1 | 1 |
| 10005473 | COG0750 | Membrane-associated protease RseP, regulator of RpoE activity | 1 | 2 |
| 10708851 | COG1030 | Membrane-bound serine protease (ClpP class) | 1 | 3 |
| 10001696 | COG1066 | Predicted ATP-dependent serine protease | 1 | 2 |
| 10000395 | COG1067 | Predicted ATP-dependent protease | 1 | 1 |
| 10006843 | COG1219 | ATP-dependent protease Clp, ATPase subunit | 1 | 2 |
| 10505542 | COG1404 | Serine protease, subtilisin family | 1 | 1 |
| 100002712 | COG1585 | Membrane protein implicated in regulation of membrane protease activity | 1 | 0 |
| 10000281 | COG2127 | ATP-dependent Clp protease adapter protein ClpS | 1 | 0 |
| 10000016 | COG3091 | Predicted Zn-dependent metalloprotease, SprT family | 1 | 0 |
| 10000883 | COG4946 | Uncharacterized N-terminal domain of tricorn protease | 1 | 8 |
| 10002503 | COG4959 | Type IV secretory pathway, protease TraF | 1 | 0 |

^1^signal peptide

**Table S3. C. Peptide depolymerisation.**

| Gene id | COG | Gene product name | Symbiont | Host |
| --- | --- | --- | --- | --- |
| 10003564 | COG2274 | ABC-type bacteriocin/lantibiotic exporters, contain an N-terminal double-glycine peptidase domain | 4 | 23 |
| 10514892 | COG0260 | Leucyl aminopeptidase | 3 | 5 |
| 10006043^1^ | COG0739 | Murein DD-endopeptidase MepM and murein hydrolase activator NlpD, contain LysM domain | 3 | 1 |
| 10000046 | COG0006 | Xaa-Pro aminopeptidase | 2 | 6 |
| 100004017 | COG0024 | Methionine aminopeptidase | 2 | 2 |
| 10004438 | COG0339 | Zn-dependent oligopeptidase | 2 | 6 |
| 10001111 | COG1362 | Aspartyl aminopeptidase | 2 | 2 |
| 10005981 | COG5009 | Membrane carboxypeptidase/penicillin-binding protein | 2 | 2 |
| 10001998 | COG0308 | Aminopeptidase N | 1 | 10 |
| 10000112^1^ | COG0405 | Gamma-glutamyltranspeptidase | 1 | 15 |
| 10000865 | COG0597 | Lipoprotein signal peptidase | 1 | 1 |
| 10000748^1^ | COG0612 | Predicted Zn-dependent peptidase | 1 | 4 |
| 10001958 | COG1376 | Lipoprotein-anchoring transpeptidase ErfK/SrfK | 1 | 0 |
| 11093861 | COG1505 | Prolyl oligopeptidase PreP, S9A serine peptidase family | 1 | 1 |
| 10001065^1^ | COG1680 | CubicO group peptidase, beta-lactamase class C family | 1 | 6 |
| 10004642^1^ | COG1686 | D-alanyl-D-alanine carboxypeptidase | 1 | 1 |
| 10001954 | COG1989 | Prepilin signal peptidase PulO (type II secretory pathway) or related peptidase | 1 | 0 |
| 10391552 | COG4099 | Predicted peptidase | 1 | 1 |
| 10003325 | COG4310 | Uncharacterized protein, cotains an aminopeptidase-like domain | 1 | 2 |
| 10068613 | COG5405 | ATP-dependent protease HslVU (ClpYQ), peptidase subunit | 1 | 0 |

^1^signal peptide

**Table S3. D. Lipid depolymerisation.**

| Gene id | COG | Gene product name | Symbiont | Host |
| --- | --- | --- | --- | --- |
| 10014563^1^ | COG2267 | Lysophospholipase, alpha-beta hydrolase superfamily | 3 | 4 |
| 10004716^1^ | COG3240 | Phospholipase/lecithinase/hemolysin | 3 | 0 |
| 10006784 | COG1752 | Predicted acylesterase/phospholipase RssA, containd patatin domain | 2 | 4 |
| 10000337 | COG1647 | Esterase/lipase | 1 | 0 |
| 10001512^1^ | COG2755 | Lysophospholipase L1 or related esterase | 1 | 1 |

^1^signal peptide
