# Supplementary figures and images for: Endozoicomonadaceae symbiont in gills of Acesta clam encodes genes for essential nutrients and polysaccharide degradation
Source: FEMS Microbiol Ecol. 2021 May 14;97(6):fiab070. doi: 10.1093/femsec/fiab070 (PMC8755941; doi:10.1093/femsec/fiab070)

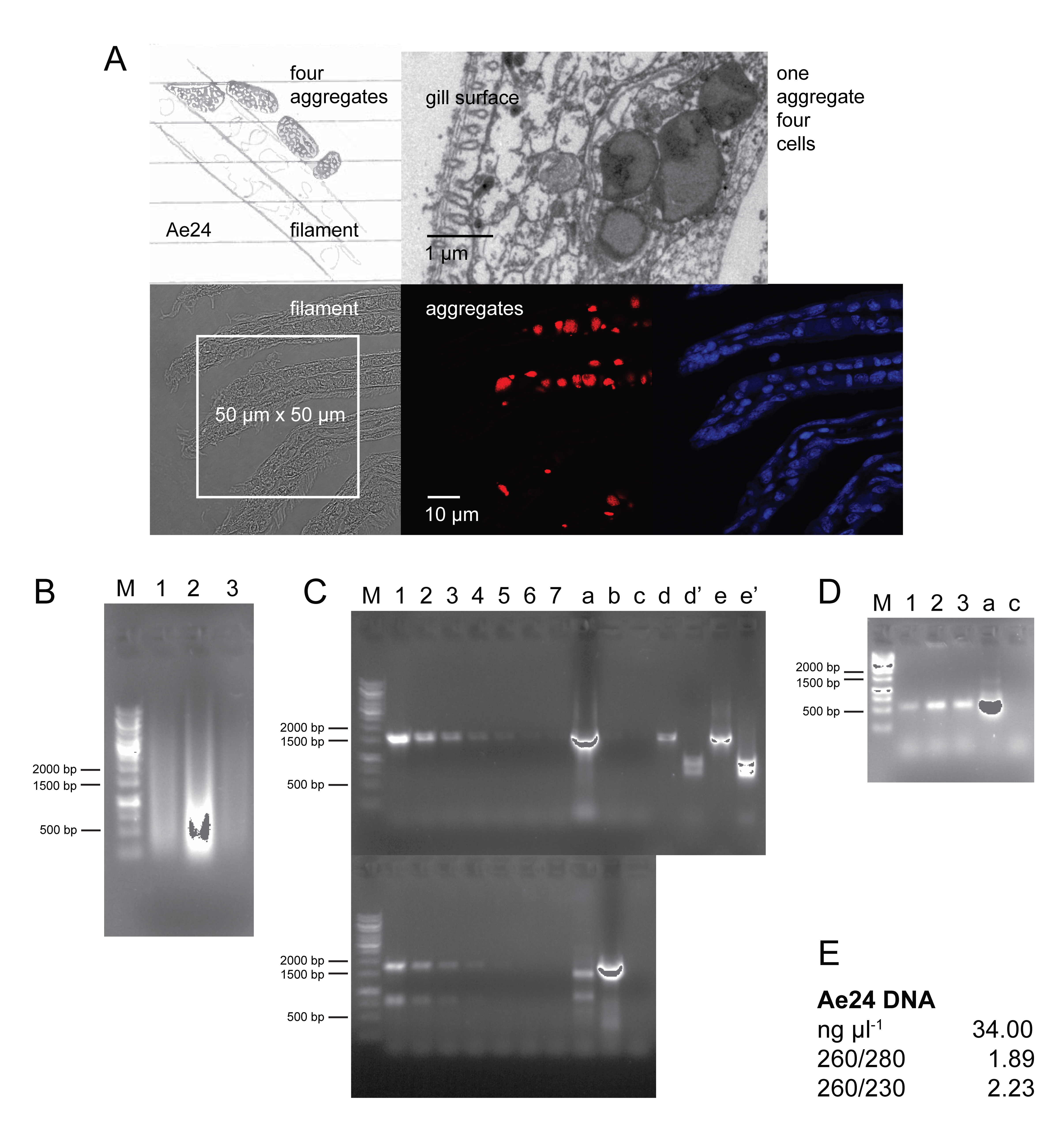

Supplement: fiab070_Supplemental_Files [file fiab070_supplemental_files.zip › FigureS1_assessment_revised_ai.tif]

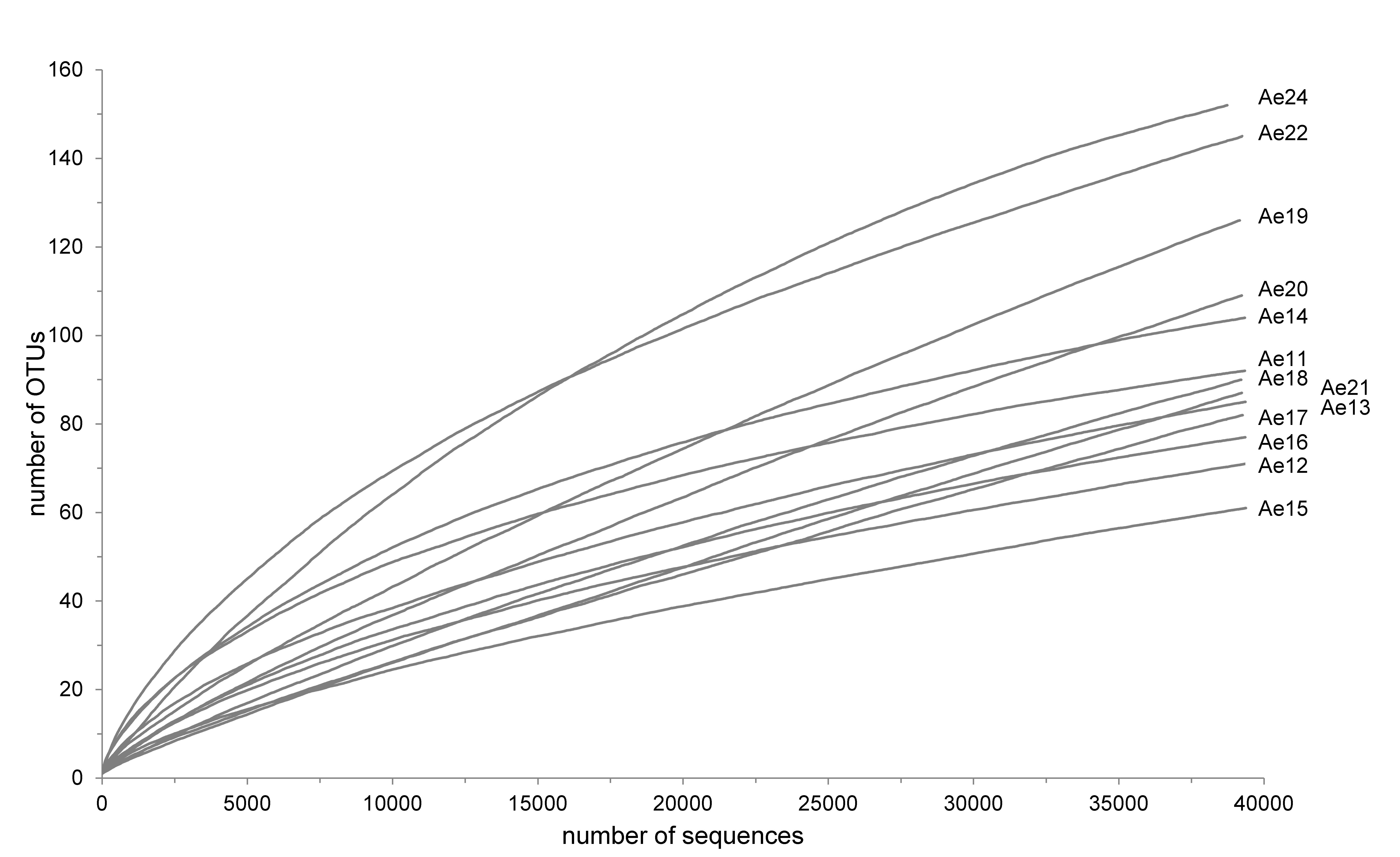

Supplement: fiab070_Supplemental_Files [file fiab070_supplemental_files.zip › FigureS2_rarefaction_R2_xls_ai.tif]

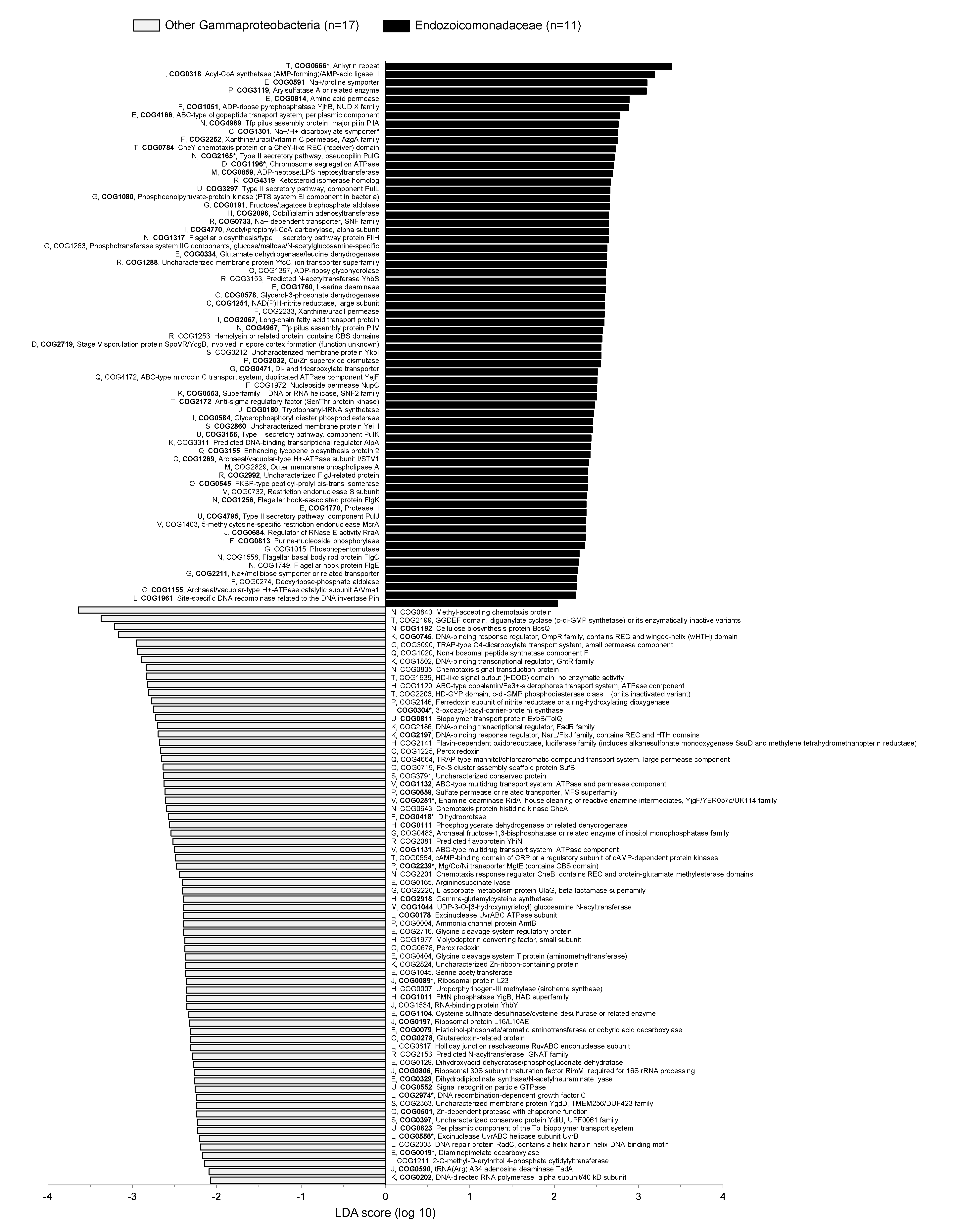

Supplement: fiab070_Supplemental_Files [file fiab070_supplemental_files.zip › FigureS3_lefse_revised_R2_xls_ai.tif]
